# Tumor malignancy by genetic transfer between cells forming cell-in-cell structures

**DOI:** 10.1038/s41419-023-05707-1

**Published:** 2023-03-13

**Authors:** Ruizhi Wang, Hao Zhong, Chenxi Wang, Xiaohui Huang, Anpei Huang, Nannan Du, Dong Wang, Qiang Sun, Meifang He

**Affiliations:** 1 Laboratory of General Surgery, The First Affiliated Hospital, Sun Yat-sen University, 58 Zhongshan 2nd Road, Guangzhou, 510080 China; 2 Department of Laboratory Medicine, The First Affiliated Hospital, Sun Yat-sen University, Guangzhou, 510080 China; 3 Laboratory of Cell Engineering, Beijing Institute of Biotechnology, Beijing, China; 4 Research Unit of Cell Death Mechanism, Chinese Academy of Medical Science, 2021RU008 Beijing, China

**Keywords:** Cancer, Mechanisms of disease

## Abstract

Cell-in-cell structures (CICs) refer to a type of unique structure with one or more cells within another one, whose biological outcomes are poorly understood. The present study aims to investigate the effects of CICs formation on tumor progression. Using genetically marked hepatocellular cancer cell lines, we explored the possibility that tumor cells might acquire genetic information and malignant phenotypes from parental cells undergoing CICs formation. The present study showed that the derivatives, isolated from CICs formed between two subpopulations by flow cytometry sorting, were found to inherit aggressive features from the parental cells, manifested with increased abilities in both proliferation and invasiveness. Consistently, the CICs clones expressed a lower level of E-cadherin and a higher level of Vimentin, ZEB-1, Fibronectin, MMP9, MMP2 and Snail as compared with the parental cells, indicating epithelial-mesenchymal transition. Remarkably, the new derivatives exhibited significantly enhanced tumorigenicity in the xenograft mouse models. Moreover, whole exome sequencing analysis identified a group of potential genes which were involved in CIC-mediated genetic transfer. These results are consistent with a role of genetic transfer by CICs formation in genomic instability and malignancy of tumor cells, which suggest that the formation of CICs may promote genetic transfer and gain of malignancy during tumor progression.

## Background

Genetically and epigenetically, tumors were heterogeneous and contained cancer cells with very different malignant potentials, in which the interaction between heterogeneous cancer cells played an important role [1]. A number of investigations suggested that the intercellular transfer of genetic information contributed to tumor heterogeneity as well as microenvironment adaption [2–4]. There were several mechanisms by which genetic information could be transmitted from one cell to another, resulting in tumor cell populations of distinct properties. Such genetic transfer, rendering acquisition of phenotypes such as tumor aggressiveness and drug resistance, could be achieved by cell fusion and phagocytosis of apoptotic bodies [3, 5, 6], whereas, the involvement of other mechanisms remains to be explored.

Cell-in-cell structures (CICs) are a type of unique cellular structure with one or more viable cells inside of another cell, which is implicated in a number of biological processes, including genome stability [7–9], immune homeostasis [10–12], inflammation [13, 14], viral infection [15–19] and the like. Based on a set of molecular machinery [20–27], CICs could be formed homotypically (same type of cells) or heterotypically (different types of cells) between cells [28], leading to the death of the internalized cells [29, 30] in an acidified lysosome [31]. CICs were most frequently documented in a variety of human tumor tissues [32–35], where CICs could promote clonal selection and tumor evolution as a mechanism of cell competition [1, 36, 37], or compromise tumor growth via mediating the in-cell killing by immune cells [38]. Accordingly, CICs and their subtypes had been shown to be an independent prognostic factor for cancer patients [39–43].

In this study, we set to explore the transferring of genetic materials between cancer cells that readily formed CICs. For this sake, two genetically marked monoclonal variants of the hepatocellular cancer cell line PLC/PRF/5 were established. The subpopulation of PLC/PRF/5-P^neo-r^ (F5-P^neo-r^) is proliferation-proficient and is resistant to G418, while the subpopulation of PLC/PRF/5-T^hygro-r^ (F5-T^hygro-r^) is metastasis-proficient and is resistant to Hygromycin B. Passaging of the CICs, formed between the above two subpopulation cells isolated by FACS, in medium contained G418 and Hygromycin B led to a new population of cells that harbor the aggressive features of their parental cells. The new derivatives are highly tumorigenic and invasive as the two parental cells did, respectively. These results are consistent with a positive role of CICs formation in tumor progression by allowing the assimilation of aggressive phenotypes from distinct coexisting subpopulations.

## Material and methods

### Cell lines and cell culture

Cell line PLC/PRF/5 (F5) was purchased from the Shanghai Cell Bank (Shanghai, China). Cells were routinely maintained inDulbecco’s Modified Eagle’s Medium (DMEM, Gibco, Gaithersbury, MD, USA) supplemented with 10% fetal bovine serum (FBS) (Gibco), which is referred to throughout as a complete culture medium. PLC/PRF/5^neo-r^ (F5^neo-r^) or PLC/PRF/5^hygro-r^ (F5^hygro-r^) cells were derived from the parental F5 cells after transfection with an expression plasmid containing the exogenous neomycin (NEO) resistance gene and red fluorescent protein (RFP) or an expression plasmid containing the exogenous hygromycin (HYGRO) resistance gene and green fluorescent protein (GFP). And then, cells were selected with selective drugs G418 (A1720, Sigma, St. Louis, MO, USA) or Hygromycin B (400051, Sigma), respectively. After several weeks of selection, stably-transfected pooled clones were selected for further use.

### Subpopulation screening

Monoclonal F5^neo-r^ or F5^hygro-r^ were obtained by the limiting dilution method. The growth rate and migration capacity of the obtained monoclones were determined by CCK-8 assay and Transwell assay. F5^neo-r^ monoclones with relatively fast growth rate were selected as F5-P^neo-r^ and F5^hygro-r^ monoclones with relatively fast migration were selected as F5-T^hygro-r^.

### Cell coculture protocol

F5-P^neo-r^ and F5-T^hygro-r^ (1:1) were mixed in suspension with or without 20 μM Y27632 for 4 h and then collected into a 6-well plate in complete culture medium with both neomycin and hygromycin B. After being cultured for 1 month, cells were then fixed with 4% paraformaldehyde, stained with 0.1% crystal violet, and photographed under an inverted microscope. Colony counts from the cocultures were statistically compared with the control results. Each treatment was performed in triplicate.

### Cell-in-cell detection

F5-P^neo-r^ and F5-T^hygro-r^ (1:1) were mixed in suspension with or without 20 μM Y27632 for the indicated time and then CICs were quantified. To obtain a cell-in-cell population, 5 × 10^6^ cells of F5-P^neo-r^ were mixed with 5×10^6^ cells of F5-T^hygro-r^ in a complete culture medium in a 10 cm plastic plate pre-coated with agarose (BY-R0100, Biowest, Nuaillé, France), After incubation for 4 h at 37 °C in a 5% CO_2_ in air atmosphere, the cells were collected, washed in PBS, and resuspended in PBS with 2 mM EDTA. CICs were counted and sorted on a BD FACScan flow cytometer (FACSAria II) (BD Biosciences, San Jose, CA) as described previously [44]. Afterward, the cells were cultured in the selection medium containing both neomycin (400 μg/mL) and hygromycin B (150 μg/mL) for 4 weeks (with fresh medium added at 3-day intervals), and monoclonal cells were picked for extended culture.

### RT- PCR and PCR

Total RNA of cell samples was extracted using TRIzol reagent (Thermo Fisher Scientific) and reverse-transcribed into cDNA using the PrimeScript RT reagent kit (Takara, Otsu, Shiga, Japan); the manufacturer’s protocol was followed. DNAs extracted from the samples were using the DNA Extraction Kit (PM0201, TSINGKE Biological Technology, Beijing, China). PCR products were resolved on 2% agarose gels containing ethidium bromide. Images were acquired using the Gel Documentation and Image Analysis System (ChampGel 5000 Plus, Sagecreation, Beijing, China). Quantitative real-time PCR (qRT-PCR) reaction was conducted on a 480 Real-time PCR System (Roche, Boston, MA, USA). All reactions were performed in triplicate, and relative gene expression was based on the 2^−ΔΔCt^ equation using GAPDH as an internal reference. Gene expression analysis (mRNA) of samples was carried out using the primer sequences (Table S1). Template cDNA was initially denatured at 95 °C for 5 min, followed by 35–40 amplification cycles consisting of denaturation at 95 °C for 1 min, primer-specific annealing for 1 min, and extension at 72 °C for 1 min. Cycles were followed by an elongation step of 72 °C for 10 min.

### Cell cycle analysis

Cells were washed three times with phosphate-buffered saline (PBS) and fixed with 75% ethanol at −20 °C overnight. After treatment with 20 μg/mL RNase A (Fermentas) at 37 °C for 30 min, the cells were resuspended in 500 μL of PBS and stained with 50 μg/mL propidium iodide in the dark for 30 min. The cells were filtered, and fluorescence was measured with a FACScan flow cytometry system (BD Biosciences).

### RNA-Seq and whole exome sequencing (WES) analysis

For RNA-Seq, total RNA was extracted using Trizol reagent (Thermo Fisher Scientific, Pittsburgh, PA, USA) following the manufacturer’s procedure. RNA-seq analysis was completed using IIIumina Hiseq 4000 (LianChuan Sciences, Hangzhou, China). Gene ontology (GO) terms for functional categorization were carried out according to molecular function, biological process, and cellular component ontologies with an E-value threshold of 10^−5^ [45]. The pathway assignments were performed by sequence searches against the Kyoto Encyclopedia of Genes and Genomes (KEGG) database and using the BLASTX algorithm with an *E*-value threshold of 10^−5^. Fragments per Kilobase of exon model per Million mapped reads values were used to measure the expression abundance of each assembled transcript. Among the five samples, a minimum of a two-fold difference in log 2 expression were considered as expression differences.

For WES, the genomic DNA (gDNA, 1 μg) was sheared to achieve target peak of 150–200 bp. After purification, size selection and adapter ligation, 10 cycles of PCR were performed for amplification of the ligation products to generate gDNA library. The prepared gDNA libraries were hybridized with Human Exome target-specific capture probe on 65 °C for 24 h. The captured libraries were sequenced on Illumina NovaSeq 6000 sequence platform using paired-end approach. BWA is utilized to perform reference genome alignment with the qualified reads contained in paired FASTQ files. And as first post-alignment processing step, Picard tools is utilized to identified and mark duplicate reads from BAM file. In the second post-alignment processing step, local read realignment is performed to correct for potential alignment errors around indels. Germline variants were called using GATK HaplotypeCaller, while single nucleotide polymorphisms and insertion-deletion polymorphisms were annotated using Annotate Variation software.

### Western blotting

As described [46], cells were lysed in radioimmunoprecipitation assay (RIPA) buffer (#89900, Thermo Fisher Scientific) to obtain the protein samples. The samples were separated by SDS-PAGE and transferred to polyvinylidene fluoride membranes (Millipore, Billerica, MA, USA), and then incubated with a 5% blocking reagent for 1 h at room temperature. Subsequently, the blots were incubated with anti-E-cadherin (Cell Signaling Technology, Danvers, MA, USA), anti-Vimentin (Cell Signaling Technology), anti-ZEB-1 (Cell Signaling Technology), anti-Fibronectin (Cell Signaling Technology), anti-MMP9 (Cell Signaling Technology), anti-MMP2 (Ser^259^) (Cell Signaling Technology), anti-Snail (Cell Signaling Technology), and β-actin (Cell Signaling Technology) at 4 °C overnight. All antibodies were diluted at a 1:1000 ratio. β-actin was used as a loading control. After washing three times with PBST, the membranes were incubated with goat anti-rabbit IgG HRP (Cell Signaling Technology) or goat anti-mouse IgG HRP (Cell Signaling Technology), diluted 1:1000, for 1 h at room temperature. Protein bands were quantified using the ECL chemiluminescence system (Tanon, Beijing, China).

### Cell proliferation assay

Cell viability was measured with the Cell Counting Kit-8 (CCK-8, Dojindo, Japan) according to the manufacturer’s instructions. Cells were plated at a density of 4 × 10^3^ cells per well in 96-well plates and incubated at 37 °C. Proliferation rates were determined at 0, 1, 2, 3, and 4 days post seeded, and measuring the absorbance of the converted dye at 450 nm. Values represent the mean ± SD of three data points from a representative experiment, and experiments were repeated three times with similar results.

### Ethynyl deoxyuridine assay

A total of 5 × 10^4^ cells/well was seeded into the 96-well plate. After 24 h, cells were cultured with DMEM media containing 5 μM Ethynyl deoxyuridine (EdU) (EdU assay kit, Thermo Fisher Scientific), 10% fetal bovine serum for 2 h. Then the cells were fixed with 4% formaldehyde at room temperature for 30 min and then treated with an Apollo reaction cocktail for 30 min. Finally, using Hoechst 33342 (Thermo Fisher Scientific) DNA staining and visualized under a fluorescent microscope.

### Transwell migration assay

Cells in each group were suspended in FBS-free medium and seeded into transwell chambers with a pore size of 8 μm (Costar, Corning, Kennebunk, ME, USA) with (invasion assay) or without (migration assay) matrigel. The lower chamber contained a medium with 10% FBS. The transwell filters were placed in a humidified incubator at 37 °C with 5% CO_2_ for 24 h. Then, cells attached to the lower surface of the membrane were fixed with 4% paraformaldehyde at room temperature for 30 min and stained with 0.5% crystal violet. The cells on the upper surface of the filter were removed by wiping with a cotton swab. Migratory cells were counted in four random microscopic fields.

### Wound-healing migration assay

Cells were seeded in confluent monolayers in six-well plates and wounds were created in confluent areas using a sterile 200 μL pipette tip after 24 h. Cell migration into the wound areas at different time points was acquired with phase contrast images and the distance cells traveled into the wound areas was measured using Image J software (National Institutes of Health, Bethesda, MD, USA). Representative images were wound-healing migration in each group and all measurements were performed in triplicate at least three times.

### Xenograft model with nude mice

SCID Beige male mice (4 weeks old) (Beijing Vital River Laboratory Animal Technology, Beijing, China) were used in the evaluation of the tumorigenicity of the derivatives in vivo. All the procedures were approved by the Institution Animal Care and Use Committee at the First Affiliated Hospital of Sun Yat-sen University. The mice were randomly divided into five groups. Each group of cells in the logarithmic growth phase was digested and the density of single cell suspension was adjusted to 5 × 10^6^/mL in PBS. Cell suspension in 100 μL was inoculated subcutaneously into the dorsal area of the mice. The tumor volume was measured and calculated based on the following formula: volume = (length × width^2^)/2. Each group included six mice.

### Statistical analysis

Statistical analysis was performed using SPSS Statistics software (version 19.0, SPSS Inc., Chicago, IL, USA). Data are reported as the mean ± SD or mean ± SEM. Two-tailed Student’s *t*-test was used to evaluate the statistical significance between the two groups, with statistical significance defined as *P* < 0.05.

## Results

### Coculture renders gene transfer between cells

**Fig. 1:**
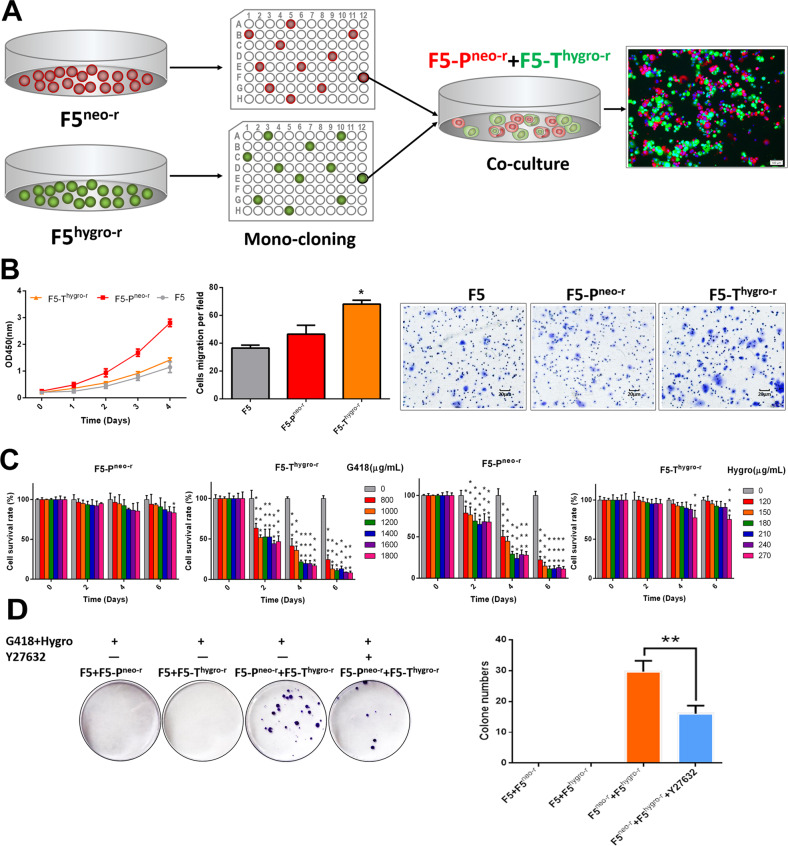
Producing dual-antibiotics-resistant clones by coculture process. **A** Schematic diagram of isolating monoclones from F5-P^neo-r^ and F5-T^hygro-r^ by limited dilution. **B** The proliferation and migration of F5, F5-P^neo-r^ and F5-T^hygro-r^ cells as determined by CCK-8 and transwell assays, respectively. Left panel: growth curve; middle and right panels: quantification and representative images of cell migration. Data are presented as the mean ± SD. Error bars denote the SD of triplicates. *P < 0.05. **C** Cell viability of F5-P^neo-r^ and F5-T^hygro-r^ cells in different concentration of G418 or hygromycin B (hygro B) as indicated. Data are presented as the mean ± SD. Error bars denote the SD of triplicates. ***P < 0.001. **D** Colony formation upon coculture in the presence of dual-antibiotics. F5 or F5-P^neo-r^ cells and F5-T^hygro-r^ were mixed 1:1 in the suspension for 24 h and then plated onto 6 cm plates for culture in complete medium containing G418 (1 mg/ml) and hygro B (150 μg/ml) in the presence or absence of Y27632 for 1 month. Colonies were stained with Crystal violet. Data are presented as the mean ± SD. Error bars denote the SD of triplicates. **P < 0.01.

By monoclonal screening, we first isolated two types of clones that are phenotype-distinct. The F5-P^neo-r^ clone, being resistant to neomycin, was highly proliferative but poorly metastatic; and the F5-T^hygro-r^ clone, being resistant to hygromycin B, was poorly proliferative but highly metastasizes (Fig. 1A, B, Supporting Fig. 1). As expected, F5-P^neo-r^ cells were sensitive to hygromycin B and F5-T^hygro-r^ cells were sensitive to neomycin (Fig. 1C). Next, F5-P^neo-r^, F5-T^hygro-r^, and their parental cells F5 were mixed with each other (1:1) in suspension for 24 h, and then plated onto 6 cm plates for culture in a complete medium with neomycin and hygromycin B for 1 month. As shown in Fig. 1D, colonies grew out from the coculture of F5-P^neo-r^ and F5-T^hygro-r^, but not from the other two cocultures (F5 + F5^neo-r^, or F5 + F5^hygro-r^), suggesting that these derivatives acquired from their coculture parental cells genetic materials that confer phenotype of drug resistance, i.e., gene transfer occurred during coculture; Interestingly, the colony formation was significantly suppressed by the treatment of Y27632 (*p* = 0.005), a well-known inhibitor of entotic CICs formation [47], suggesting that formation of CICs might contribute to the gene transfer.

### Gene transfer upon cell-in-cell formation

**Fig. 2:**
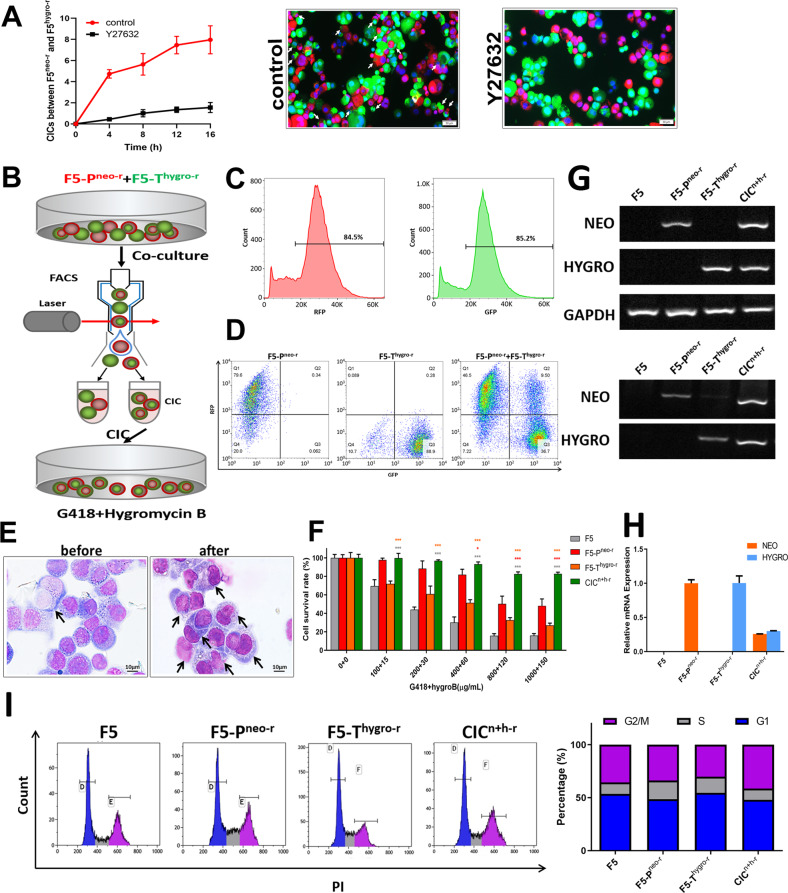
Evidence for genetic information transferred from both parental cells by cell-in-cell process. **A** CICs formation between F5^neo-r^ cells and F5^hygro-r^ for indicated time in medium with or without Y27632. **B** Schematic diagram of cell-in-cell process from F5-P^neo-r^ and F5-T^hygro-r^ by FACS assay. **C** Detecting the RFP or GFP fluorescence signal of F5-P^neo-r^ and F5-T^hygro-r^ cells by Flow cytometry. **D** Enrichment of CICs formed between F5-P^neo-r^ and F5-T^hygro-r^ cells by Flow cytometry. **E** Representative images of F5-P^neo-r^ and F5-T^hygro-r^ cell populations before and after sorting by flow cytometry staining with Giemsa. **F** Detecting cell survival rate of F5, F5-P^neo-r^, F5-T^hygro-r^, CIC^n+h-r^ cells in different concentration of G418 and hygro B in combination. **G** Detecting antibiotic resistance markers (NEO or HYGRO) and GAPDH from RNAs (upper panel) or DNAs (lower panel) extracted from F5, F5-P^neo-r^, F5-T^hygro-r^ and CIC^n+h-r^ cells by PCR. **H** The expression of NEO or HYGRO genes of F5, F5-P^neo-r^, F5-T^hygro-r^ and CIC^n+h-r^ cells detected using qRT-PCR. **I** Flow cytometric analysis of F5, F5-P^neo-r^, F5-T^hygro-r^, CIC^n+h-r^ cells for DNA content by propidium iodide (PI) staining.

To examine the roles of CICs formation in gene transfer, we first confirmed that CICs formation between F5-P^neo-r^ and F5-T^hygro-r^ cells could be efficiently inhibited by the treatment of Y27632 (Fig. 2A), which was consistent with a previous report [29, 48]. Then, F5-P^neo-r^ cells expressing RFP cocultured in suspension with F5-T^hygro-r^ cells expressing GFP were sorted by fluorescence-activated cell sorting (FACS) for double-positive cells representing CICs (Fig. 2B, C). The CICs efficiency represented by double-positive cells was about 9.5% for 6 h-incubation (Fig. 2D, E). Subsequently, the CICs population were cultured in a medium containing both antibiotics (NEO and HYGRO) for 1 month. The new colonies successfully grew out with a frequency of approximately 1/10^4^. Cell survival assay showed that the new derivative (CIC^n+h-r^) was resistant to both antibiotics (Fig. 2F). To confirm that the derivatives contained and expressed both drug resistance markers, RT-PCR was performed to amplify the cDNA of neomycin or hygromycin resistant genes, which successfully detected both genes’ expression in the dual-resistant derivative CIC^n+h-r^ (Fig. 2G, upper panel). Similar results were obtained when PCR was performed on DNA from CIC^n+h-r^ cells (Fig. 2G, lower panel). Finally, expression quantification of the two resistant genes by RT-PCR indicated that the CIC^n+h-r^ cells expressed about 1/4 of the two drug-resistantnt genes (26% for NEO and 28% for HYGRO) as compared with their parental cells (Fig. 2H). Flow cytometry analysis indicated that both the parental cells (F5, F5-P^neo-r^ or F5-T^hygro-r^) and new derivatives (CIC^n+h-r^) were not significantly different in the composition of DNA content (Fig. 2I), implying that the CIC^n+h-r^ cells were not derived from parental cell–cell fusion, the latter frequently result in increased genomic DNA content. Together, these results are consistent with the notion that gene transfer occurred during the CICs process.

### Gain of malignant phenotypes in CICs derivatives

**Fig. 3:**
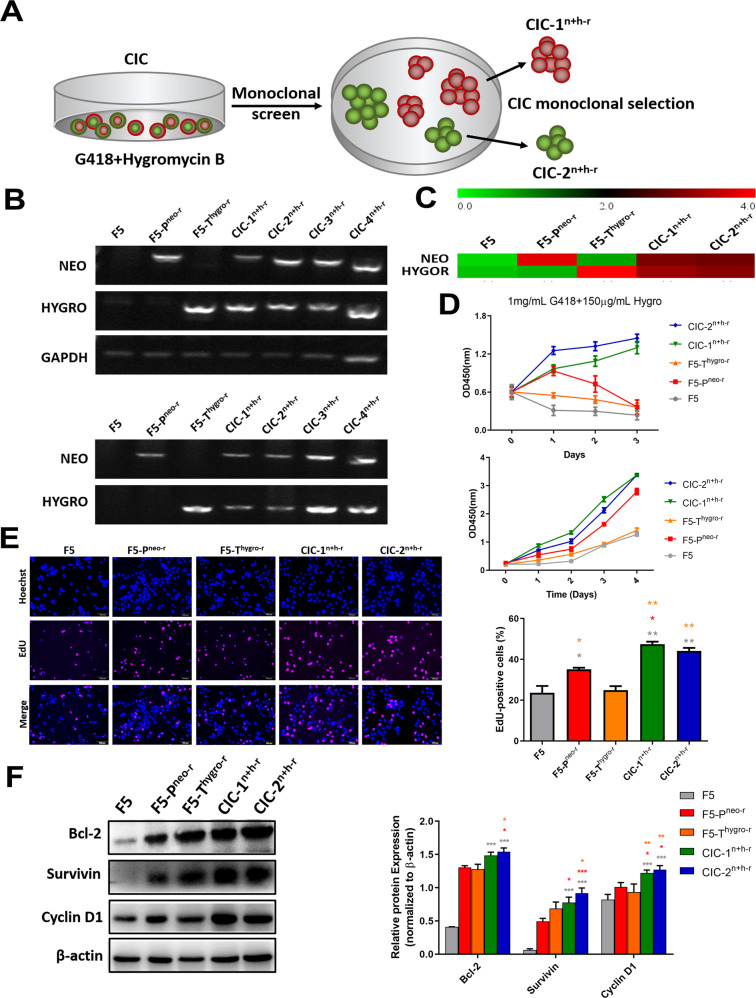
The dual-antibiotic-resistant CIC derivative exhibited significant proliferation ability. **A** Schematic diagram of CICs monoclonal in medium with both G418 and hygromycin B. **B** Detecting the neomycin (NEO) or hygromycin resistance marker (HYGRO) from RNAs (upper panel) and DNAs (lower panel) extracted from F5, F5-P^neo-r^, F5-T^hygro-r^ and several CIC monoclonals by PCR. **C** The heatmap reflecting log_2_ (expression of NEO and HYGRO target genes) in F5, F5-P^neo-r^, F5-T^hygro-r^ and CIC^n+h-r^ cells. **D** The proliferation of F5, F5-P^neo-r^, F5-T^hygro-r^ and CIC^n+h-r^ cells with (upper panel) or without (lower panel) 1 mg/mL G418 and 150 μg/mL hygro B at different time points using CCK-8 assays. **E** The proliferation of F5, F5-P^neo-r^, F5-T^hygro-r^ and CIC^n+h-r^ cells by Edu staining assay. **F** The expression of Bcl-2, Survivin, Cyclin D1 and β-actin was used as control in F5, F5-P^neo-r^, F5-T^hygro-r^ and CIC^n+h-r^ cells Western blot analysis (left). The bands of proteins were quantified by densitometry and normalized to β-actin protein (right). * *P* < 0.05, ** *P* < 0.01, *** *P* < 0.001.

Considering that gene transfer during the CICs process endowed the phenotype of drug resistance, it is therefore anticipated that other phenotypes associated with tumor malignancy might be transferred as well. To test this idea, we first isolated several monoclones from the CICs derivatives as depicted in Fig. 3A. PCR on RNA or DNA extracted from these clones confirmed the presence and expression of drug resistance genes, indicating successful gene transfer (Fig. 3B), which was further confirmed by RNA-seq analysis of two CICs clones (CIC-1^n+h-r^, CIC-2^n+h-r^) (Fig. 3C). Next, cell growth was accessed for the two CICs clones by CCK-8 assays in the presence of both antibiotics (1 mg/mL G418 and 150 μg/mL Hygromycin). As illustrated in the upper panel of Fig. 3D, the CICs colonies (CIC-1^n+h-r^ and CIC-2^n+h-r^) could grow efficiently despite that the growth of parental cells was significantly suppressed. Importantly, the growth advantage of CICs clones is maintained even in the absence of antibiotics (Fig. 3D, lower panel), which is correlated with enhanced Edu incorporation (Fig. 3E) and the upregulated expression of cell cycle promoter cyclin D1 and pro-survival proteins (Bcl-2 and survivin) (Fig. 3F), suggesting that gene transfer confers a growth advantage to the CICs derivatives.

**Fig. 4:**
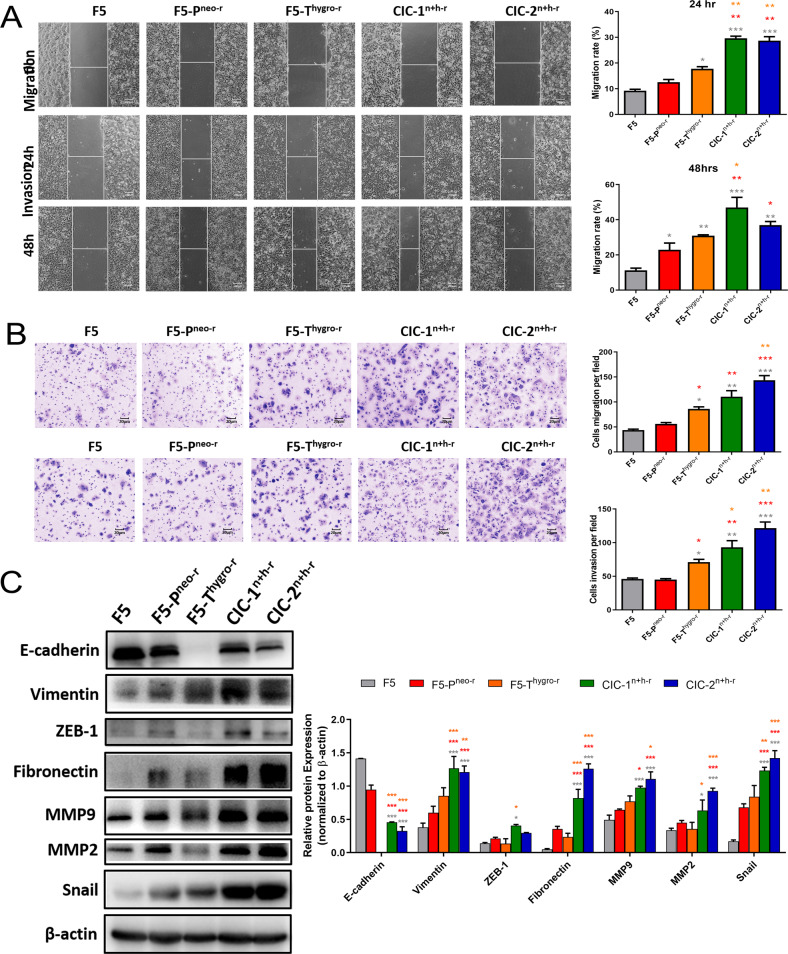
The dual-antibiotic-resistant CIC derivative cells exhibited more metastatic potential. **A** Representative images of wound healing of F5, F5-P^neo-r^, F5-T^hygro-r^, CIC-1^n+h-r^ and CIC-2^n+h-r^ cells. Histograms depict the average migrated distance. **B** Detecting the migration (upper panel) and invasion (lower panel) ability of F5, F5-P^neo-r^, F5-T^hygro-r^ CIC-1^n+h-r^ and CIC-2^n+h-r^ cells. Four randomly selected fields were captured and quantitation was presented in the graph. Representative micrographs are also shown (200 × magnification). **C** The expression of E-cadherin, Vimentin, ZEB-1, Fibronectin, MMP9, MMP2, Snail and GAPDH was used as control in F5, F5-P^neo-r^, F5-T^hygro-r^, CIC-1^n+h-r^ and CIC-2^n+h-r^ cells by Western blot analysis(left). The bands of proteins were quantified by densitometry and normalized to β-actin protein (right). All data are showed as the mean ± S.D. of three independent experiments, * *P* < 0.05, ** *P* < 0.01, *** *P* < 0.001 * *P* < 0.05, ** *P* < 0.01, *** *P* < 0.001.

Since one of the parental cells was highly invasive, we next investigate whether the two CICs clones inherited the related phenotypes. As shown in the wound-healing assay in Fig. 4A, the two CICs clones migrated more significantly than their parental cells, which was confirmed in the transwell migration assay (Figure B, upper panel). Moreover, in the transwell invasive assay, the CICs clones also penetrated across the matrigel significantly more than their parental cells did (Fig. 4B, bottom panel). In line with enhanced migration and invasiveness, the CICs clones expressed a relatively lower level of E-cadherin and a higher level of Vimentin, ZEB-1, Fibronectin, MMP9, MMP2 and Snail as compared with the parental cells (Fig. 4C). The altered molecular expression pattern resembled that occurred during the process of Epithelial-Mesenchymal Transition (EMT), which is critical for cancer cell migration and invasion. Thus, the above data are consistent with a gain of malignant phenotypes, such as enhanced cell growth, migration and invasiveness, by the derivatives during the CICs process.

### Enhanced tumorigenicity of CICs derivatives in vivo

**Fig. 5:**
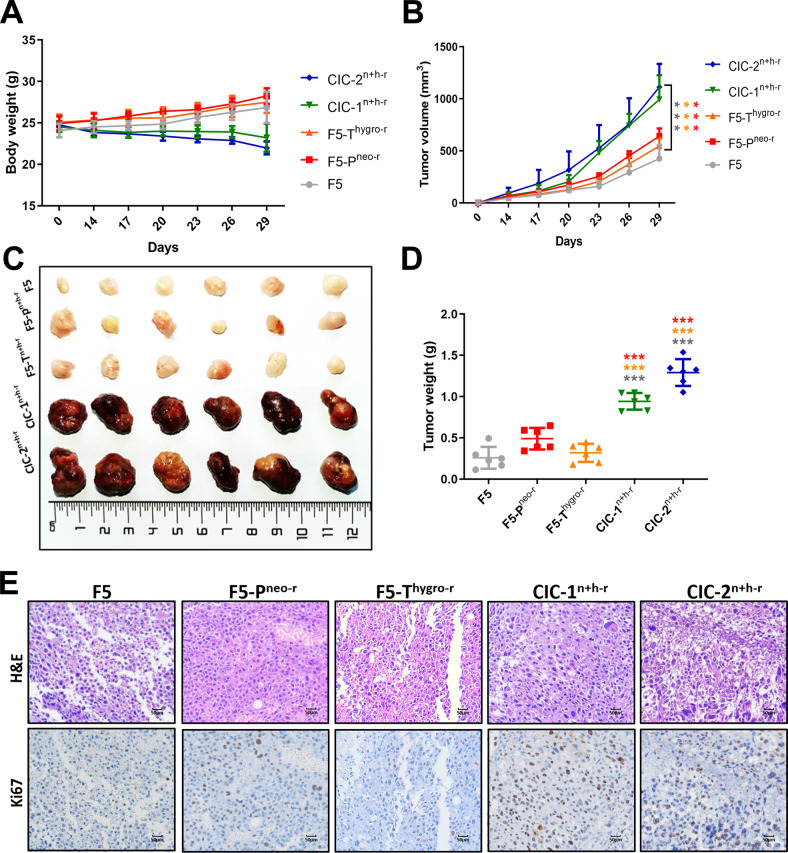
The effect of dual-antibiotic-resistant clones CIC-1^n+h-r^ and CIC-2^n+h-r^ cells on xenograft tumor growth. **A** Mice body weight with time course. **B** Tumor growth of F5, F5-P^neo-r^, F5-T^hygro-r^, CIC-1^n+h-r^ and CIC-2^n+h-r^ with time course. **C** Visual comparison of F5, F5-P^neo-r^, F5-T^hygro-r^, CIC-1^n+h-r^ and CIC-2^n+h-r^ cells dissected tumor tissues. **D** The tumor weight of F5, F5-P^neo-r^, F5-T^hygro-r^, CIC-1^n+h-r^ and CIC-2^n+h-r^ at the indicated time point. **E** HE staining and Ki67 in subcutaneous xenografts in nude mice.

Furthermore, to examine whether the malignant phenotypes gained at cellular level could be transformed into tumorigenicity in vivo, the CICs clones (CIC-1^n+h-r^ and CIC-2^n+h-r^) were injected subcutaneously on the back of SCID Beige nude mice (*N* = 6) and mice were observed for 4 weeks for tumor formation. While the body weight of the mice tended to decrease (Fig. 5A), the xenograft tumors grew significantly faster in the CICs clone groups in comparison with the control groups, as manifested by larger tumor volume (Fig. 5B, C) and heavier tumor weight (Fig. 5D). Histological staining showed that the positive expression of Ki67 protein in the CICs clone groups were significantly higher than those in control groups (Fig. 5E). This result indicated that the tumorigenicity of the tumor cells increased after CICs process, suggesting a positive role of CICs-associated gene transfer in tumor progression.

### Gene expression profiling of CICs derivatives

**Fig. 6:**
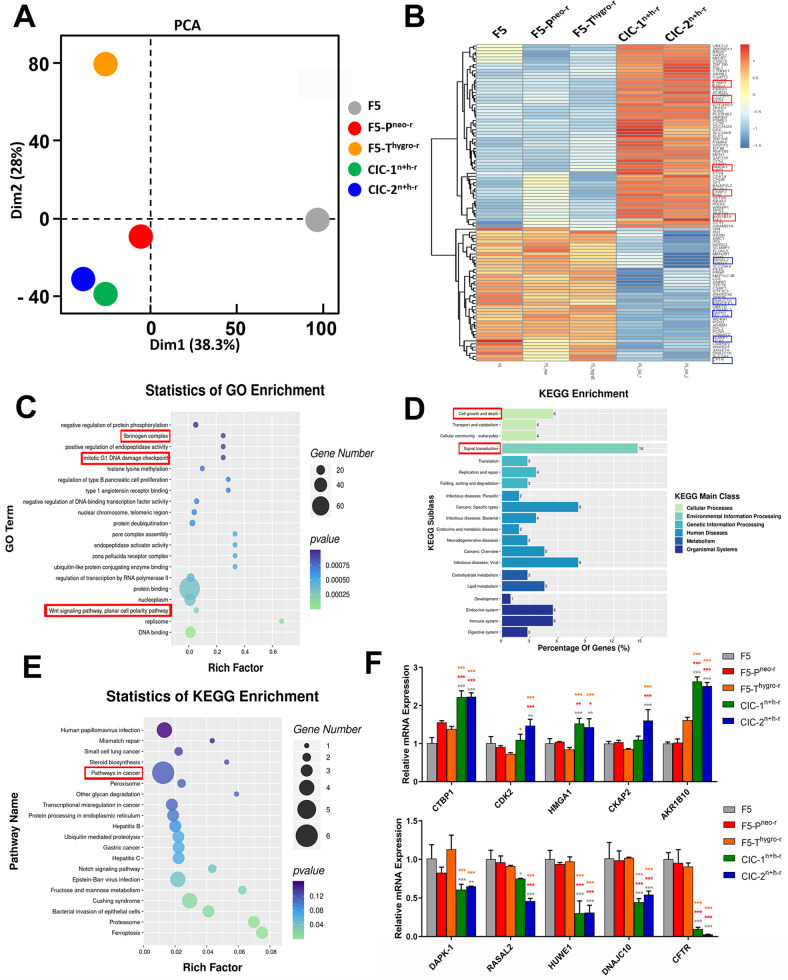
The transcriptional changes of the dual-antibiotic-resistant derivative CIC^n+h-r^ compared to parental cells. **A** The principle component analysis of F5, F5-P^neo-r^, F5-T^hygro-r^, CIC-1^n+h-r^ and CIC-2^n+h-r^ cells. **B** The colors of the heatmap reflect log_2_ (Differentially expressed genes) in F5, F5-P^neo-r^, F5-T^hygro-r^, CIC-1^n+h-r^ and CIC-2^n+h-r^ cells. **C** GO term analysis of differently expression genes (DEGs). **D** KEGG pathway analysis of signal transduction pathways involved in CIC-1^n+h-r^ and CIC-2^n+h-r^ cells compared to F5, F5-P^neo-r^, F5-T^hygro-r^ cells. **E** Statistics of KEGG enrichment of CIC-1^n+h-r^ and CIC-2^n+h-r^ cells compared to F5, F5-P^neo-r^, F5-T^hygro-r^ cells. **F** Relative mRNA expression of differently expression genes in CIC-1^n+h-r^ and CIC-2^n+h-r^ cells compared to F5, F5-P^neo-r^, F5-T^hygro-r^ cells.

To explore the molecular changes underlying the malignant phenotypes gained by the CICs clones, the gene expression profiles were analyzed by RNA-seq. Principle component analysis (PCA) showed that the two CICs clones tightly clustered with each other, which showed striking difference to their original parental cell F5, followed sequentially by F5-P^neo-r^ and F5-T^hygro-r^ (Fig. 6A). Analysis of differentially expressed (DE) genes by the cutoff of fold changes ≥ 2 and p ≤ 0.05 identified 102 genes, with 59 upregulated and 43 downregulated, respectively, between CICs clones and their parental cells (Table S2). The result was visualized via heatmap in Fig. 6B. Enrichment analysis was performed for all GO terms annotated to the significant DE genes to determine the relative degree of GO term enrichment across all categories (Fig. 6C). The results indicated a set of enriched processes related to cancer malignancy, such as those associated with fibrinogen complex, mitotic G1 DNA damage checkpoint and Wnt signaling pathway. Similarly, KEGG enrichment analysis characterized the enriched biological functions, such as “cell growth and death” and “signal transduction” (Fig. 6D). Of note, “pathways in cancer” was identified in the top 20 significantly enriched KEGG pathways (Fig. 6E). Moreover, the expression of 10 DE genes were further validated by qRT-PCR, including CTBP1, CDK2, HMGA1, CKAP2 and AKR1B10 that are upregulated, and DAPK1, RASAL2, HUWE1, DNAJC10 and CFTR that are downregulated in the CICs clones (Fig. 6F). The overexpression of CDK2, CKAP2, CCT3 were known to promote cell proliferation, and upregulation of UBE2J2, or downregulation of CFTR, RASAL2, DAPK1, HUWE1 could promote EMT. Thus, these molecular changes set a genetic basis for the malignant phenotypes of CICS derivatives gained during the CICs process.

### Detection of gene transfer in CIC derivatives by whole-exome sequencing (WES)

**Fig. 7:**
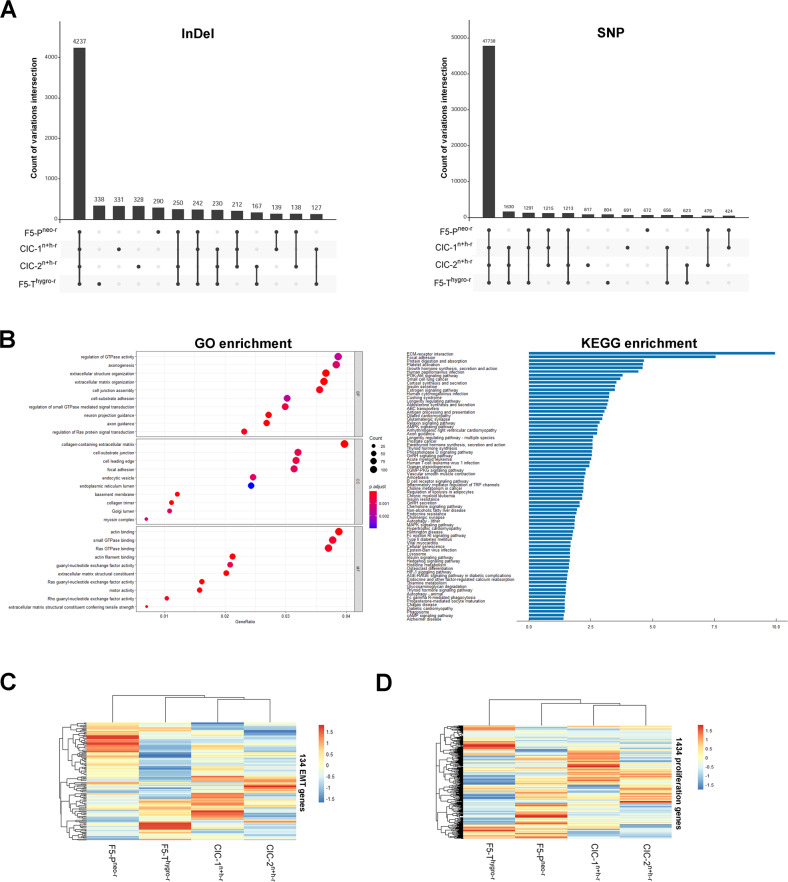
Bioinformatic analysis of gene transfer in CIC derivatives. **A** Upset plot of germline InDels and SNPs identified in F5-P^neo-r^, F5-T^hygro-r^, CIC-1^n+h-r^ and CIC-2^n+h-r^ cells. The intersection is represented by the bottom plot, and their occurence is shown on the top barplot. The black dots connected by lines show which variations set is part of an intersection. The number of shared variations (6051) by one or both CIC derivatives and parental cells is summed up by intersection amounts highlighted by red boxes. **B** GO and KEGG enrichment analysis of 3796 genes harboring above 6051 shared variations. Cluster analysis and heatmap plotting of the (**C**) EMT- and (**D**) proliferation-associated gene expression measured by RNA-seq in F5-P^neo-r^, F5-T^hygro-r^, CIC-1^n+h-r^ and CIC-2^n+h-r^ cells.

To further investigate whether gene transfer by CICs contribute to the malignancy acquisition, a WES assay was performed with the parental and daughter cells. The unique germline variations between the two parental cells were used as DNA makers to detect the potential genomic regions that transferred from parental cells to daughter cells. This analysis showed that a total of 6051 variations (including SNP and InDel) located in 3796 genes, were shared by one or both CIC derivatives and parental cells (Fig. 7A, Supporting Table), implying that DNA transfer might occur in these gene loci of daughter cells. Importantly, enrichment analysis revealed that these genes were significantly enriched in growth- and metastasis-related process and pathways, including small GTPase pathway, cell junction, extracellular matrix organization, Ras signaling and so on (Fig. 7B). In depth, these transfer-occurred genes contain 134 EMT genes and 1434 proliferation genes (Supporting Table), of which the expression levels in the four cell lines were subjected to cluster analysis. The clustering results showed that the expression patterns of the 134 EMT genes in daughter cells are significantly more similar with the highly metastatic parental cell line F5-T^hygro-r^ (Fig. 7C), meanwhile the daughter cells displayed more similar expression patterns of the 1434 proliferation genes with highly proliferative F5-P^neo-r^ but not F5-T^hygro-r^ cells (Fig. 7D). These data highly suggested that the DNA transfer occurred in EMT and proliferation genes by CIC endowed daughter cells with the characteristic gene expression of parental cells, thereby promoting malignancy gain of CIC derivatives.

## Discussion

Recent evidence indicated that alternative mechanisms, such as CICs process, might also render cells the ability to escape cell cycle control, tissue invasion, and metastasis [9, 49–51]. Herein, we reported that CICs formation between tumor cells resulted in cell clones associated with genetic transfer and gain of malignancy, which uncovered a novel route whereby CICs formation promotes tumor progression. In the present study, we found that the new clones acquired both drug resistance genes of NEO and HYGRO from their parental cells, along with new characteristics, such as increased proliferation and invasiveness. These results support that tumor cells may achieve genetic transfer through CICs process. This provided a new mechanism allowing rapid tumor evolution, which is consistent with the pivotal roles of entotic CICs formation in clonal selection and tumor evolution as proposed previously [1, 36, 37, 52]. Given the extensive genetic heterogeneity among the cancer cells in hepatocellular tumors [53], it is conceivable that this process would contribute to the acquisition of multiple genetic characteristics by a defined group of tumor cells. In fact, association with CICs was a strong predictor of shorter postoperative survival of patients with certain types of cancer such as pancreatic adenocarcinoma [39, 51], which may also help explain the preferential development of aggressive cancer types after therapy in some cancer patients [39].

It was demonstrated that human cancer cells could exchange of genetic information in several studies [3, 6, 54, 55]. Previous research demonstrated that cell fusion mediated horizontal gene transfer resulting in information of reprogrammed somatic cell hybrids [56–58]. It has been reported cell fusion between transformed cells and normal stem cells and might be important for reprogrammed somatic cell hybrids formation with a highly metastatic self-renewing phenotype [59]. Another documented mechanism that up-taken of apoptotic bodies by phagocytosis was mediated by horizontal transfer of apoptotic DNA to normal cells resulting in senescence and cell cycle arrest [3, 54]. A similar experiment was reported where EBV-encoded genes EBER and EBNA1 could be transferred by apoptotic bodies EBV-carrying B lymphocytes to the recipient cells without the receptor for the virus at a high frequency [54]. Based on our results, we proposed that CIC process described a new means of passing genetic information directly from one cell to another cell that formed CICs, and that this process had the potential to mediate the passage of phenotypic characteristics between cancer cells within a tumor.

EMT is an evolutionarily conserved process that occurs during development and may also be involved in cancer. Previous studies showed that several genes encoding transcription factors, including Twist, Snail and Slug, governed EMT [60]. During EMT, epithelial cells losing intercellular junctions penetrate into the extracellular matrix-rich compartment. E-cadherin is a key component of adherens junctions and the suppression of E-cadherin and a switch to the expression of mesenchymal cadherins, such as N-cadherin, are associated with tumor invasion [61]. Herein, we showed that CICs process occurred between tumor cells might promote EMT of the new clones through genetic transfer.

By WES and RNA-seq analysis, the present studies preliminarily identified potential genes involved in CIC-mediated genetic transfer and speculated that the genetic transfer during the CICs process endowed the phenotype of malignant growth and metastasis. In addition, according to our data, a large set of genes but not one or several genes might integrate the genome of daughter cells via CIC process and contribute to growth enhancement and EMT transition, prompting the universality of genetic transfer by CICs formation, which is consistent with previous studies [62, 63]. It is also important to point out that the WES assay displays relatively poor performing and low resolution in detecting DNA transfer between homogeneous cells. New technology should be developed to identify the detailed DNA content in CIC-mediated genetic transfer. And further investigations should focus on the mechanisms by which CIC-mediated genetic transfer regulates gene expression.

## Conclusions

Together, this study reported that tumor cells may achieve genetic transfer to gain of malignancy during tumor progression, which is dependent on the formation of CICs. Moreover, CICs process may contribute to genomic instability and creation of highly metastatic cells. This provides a new mechanism allowing rapid tumor evolution, which is consistent with the pivotal roles of CICs formation in clonal selection and tumor evolution in hepatocellular carcinoma.

## Supplementary information

supplemental materials

aj-checklis

Original Data File

supportting table

Table S1

Table S2

## Data Availability

The datasets generated for this study are available on request to the corresponding author.
