# Supplementary material for: Tumor malignancy by genetic transfer between cells forming cell-in-cell structures
Source: Cell Death Dis. 2023 Mar 13;14(3):195. doi: 10.1038/s41419-023-05707-1 (PMC10011543; doi:10.1038/s41419-023-05707-1)

Supporting Figure legends

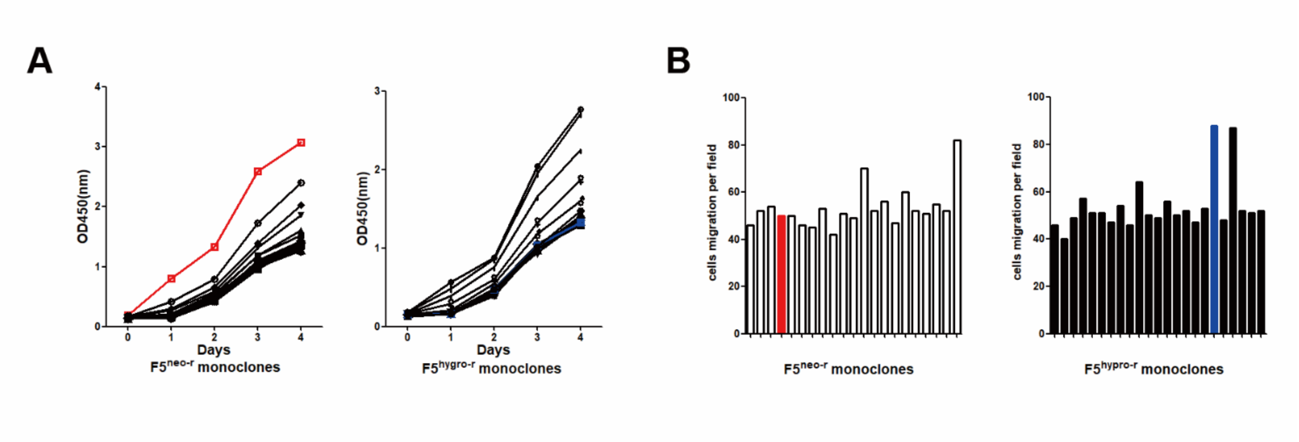

Supporting Figure 1 The growth rate (A) and migration capacity (B) of F5^{neo-r} and F5$^{hygro-r}$ monoclones. The selected monoclones F5-P^{neo-r} and F5-T$^{hygro-r}$ were highlight as red and blue respectively.

WT1
WNT5B
VWCE
UHRF1
TRPS1
TP73
TNS1
TJP1
STAT5B
STAT5A
SMAD3
SEMA7A
ROS1
RNH1
PTP4A3
PLXND1
PITPNM1
PIK3R3
PARP1
NRP2
NOTCH4
NOTCH3
NFKBIA
NFKB1
MUC4
MUC2
MUC16
MTOR
MMP11
MICAL2
MICA
MAP4K4
MAP3K7
LRRFIP1
LMNA
LATS1
LAMA5
KL
KCNH1
JAG2
ITGB6
ITGB4
ITGA3
IRS2
IGFBP7
HDAC9
HDAC5

GEMIN2
FOXM1
FOSL1
FERMT1
ETS2
ERN1
EPHB2
EPHA2
EML4
DDR1
CUL4A
CSPG4
CRK
CREBBP
COL8A2
CDKN2A
CDKN1A
CCND1
CCL3
CAMK1D
C5AR1
AXIN2
AXIN1
ARRB1
ANPEP
ALX4
ALK
ACVR1
ABCB1
VGLL4
TPBG
TNXB
TGM2
TGFBR3
TACC3
SATB1
RGCC
PSME3
PLXND1
PADI4
NOTCH4
MUC4
MUC2
MUC16
MMP14
MARVELD3
L1CAM

IRS2
HOOK1
GRN
GLI2
FSCN2
FN1
FASN
ESRRB
CUX1
CSPG4
CCR7
BATF2
TRIM62
TRAP1
TNXB
TNS1
TMPRSS4
SQSTM1
REPIN1
RBFOX3
PTP4A3
PDGFRB
NRP2
MUC2
MARVELD3
MAPK14
MALAT1
LMNA
LAMA5
IL27
HDAC4
FSCN2
FN1
FERMT2
EXOC7
EXOC4
DKK3
DAB2IP
CSPG4
CRIM1

Supplemental: full Western blots

1.

Bcl-2: 26 kDa

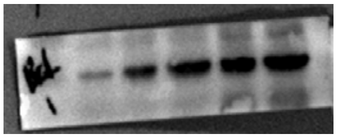

Survivin: 16kDa

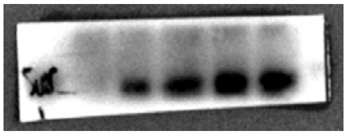

Cyclin D1: 34kDa

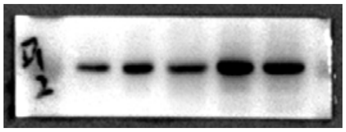

β-actin: 41kDa

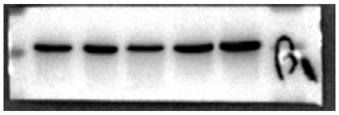

E-cadherin: 90kDa

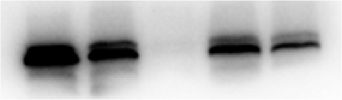

Vimentin: 53kDa

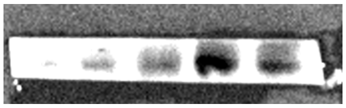

ZEB-1: 200kDa

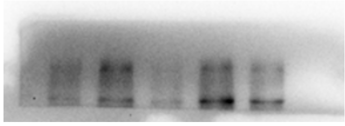

Fibronectin: 263kDa

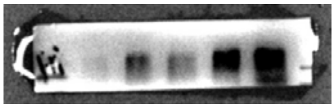

MMP9: 78 kDa

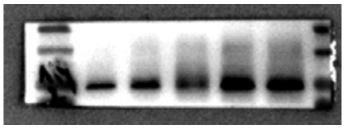

MMP2: 70 kDa

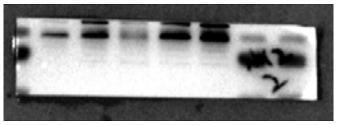

Snail: 29 kDa

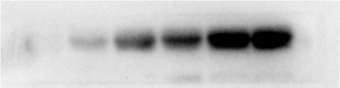

β-actin: 41kDa

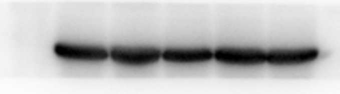

Supplement: Supplementary file 1 — supplemental materials [file 41419_2023_5707_MOESM1_ESM.pdf]
