# Supplementary figures and images for: Tumor malignancy by genetic transfer between cells forming cell-in-cell structures
Source: Cell Death Dis. 2023 Mar 13;14(3):195. doi: 10.1038/s41419-023-05707-1 (PMC10011543; doi:10.1038/s41419-023-05707-1)

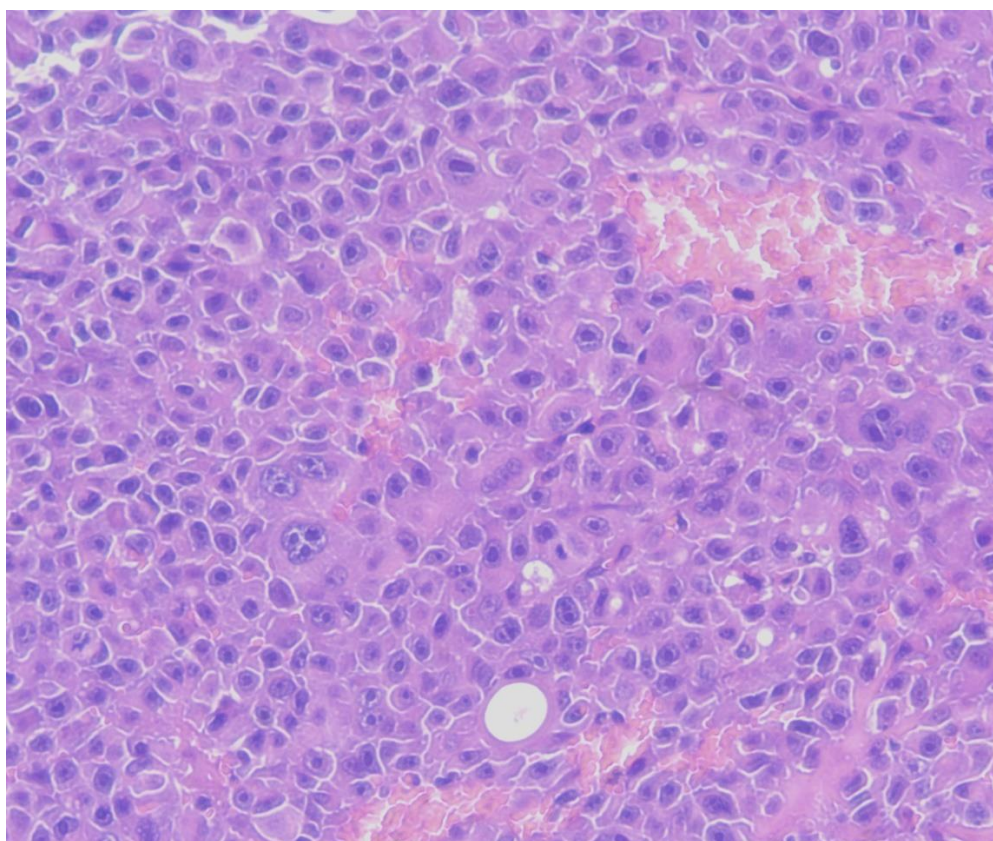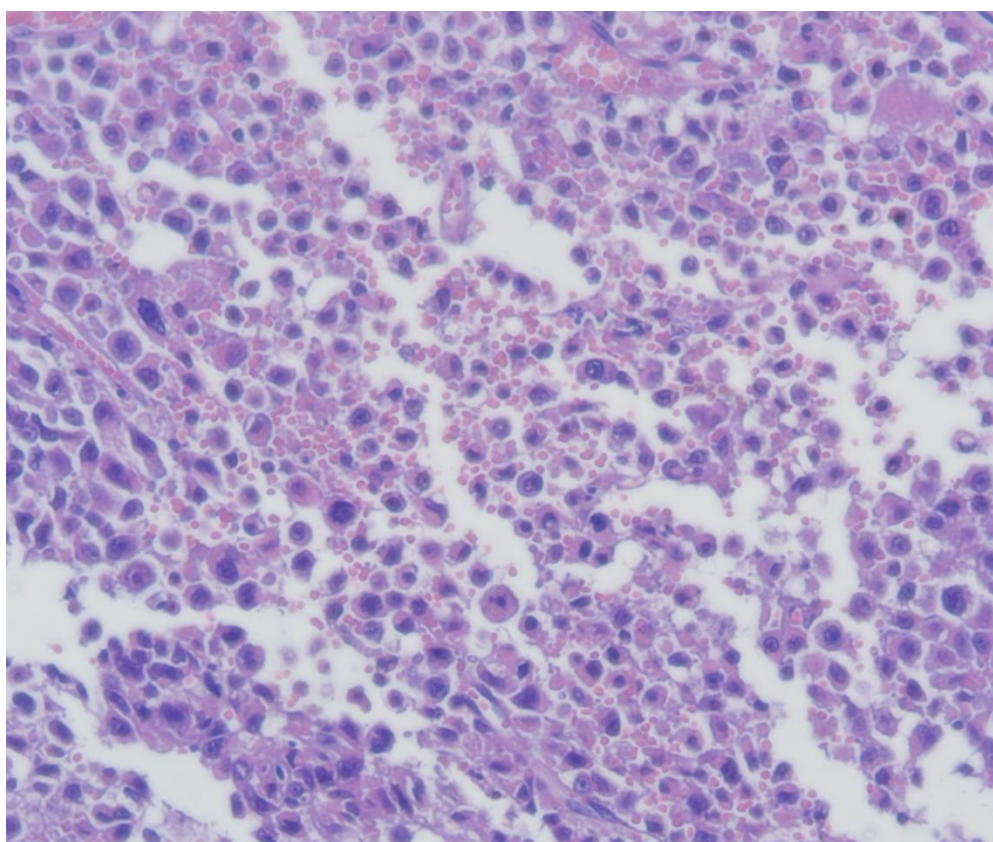

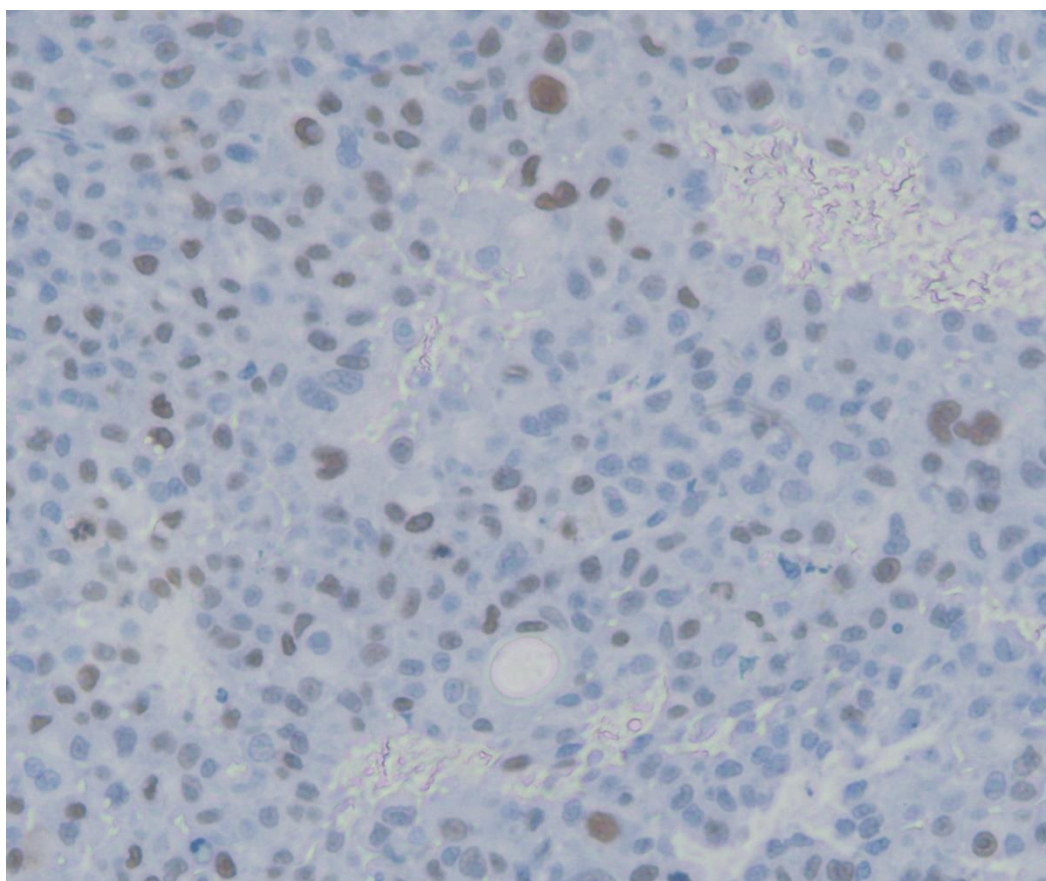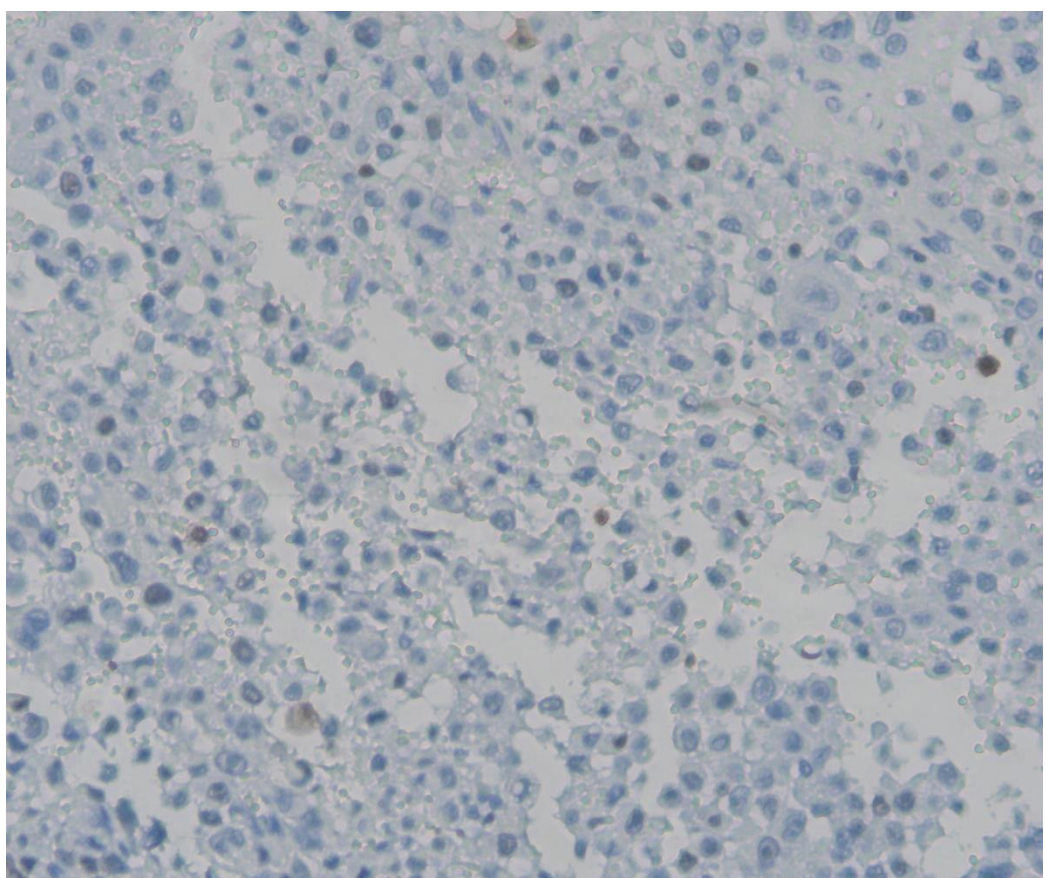

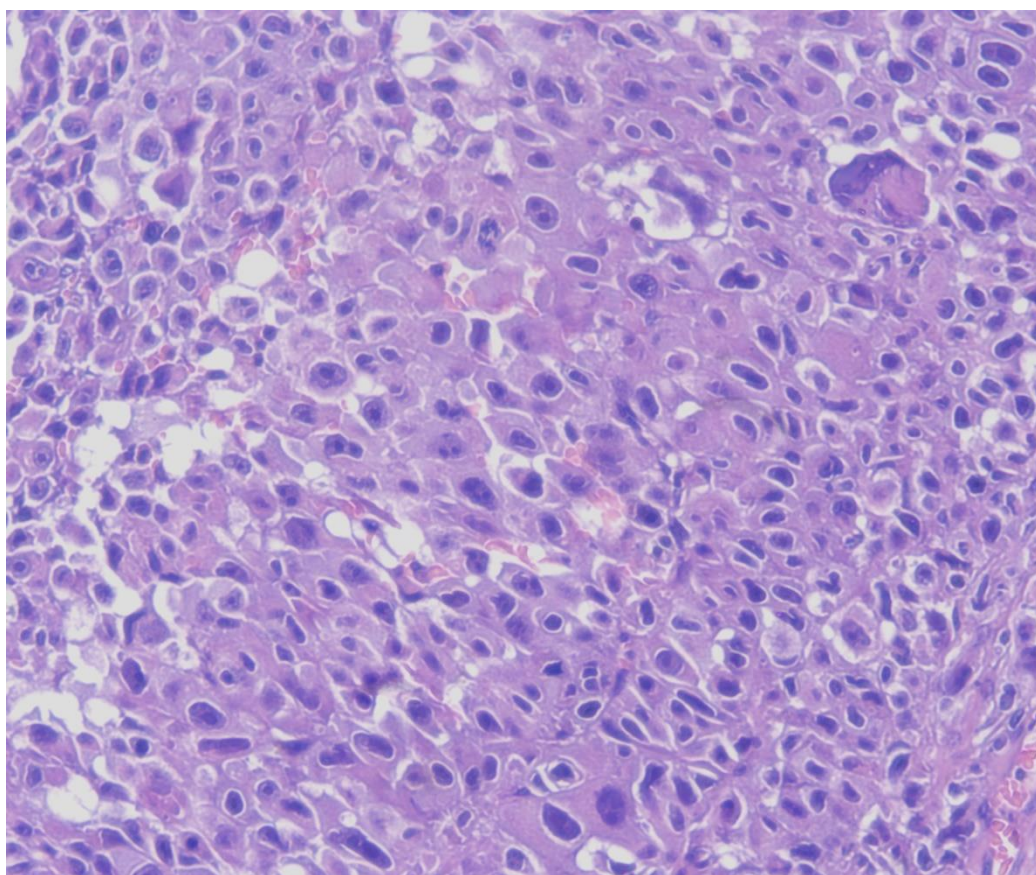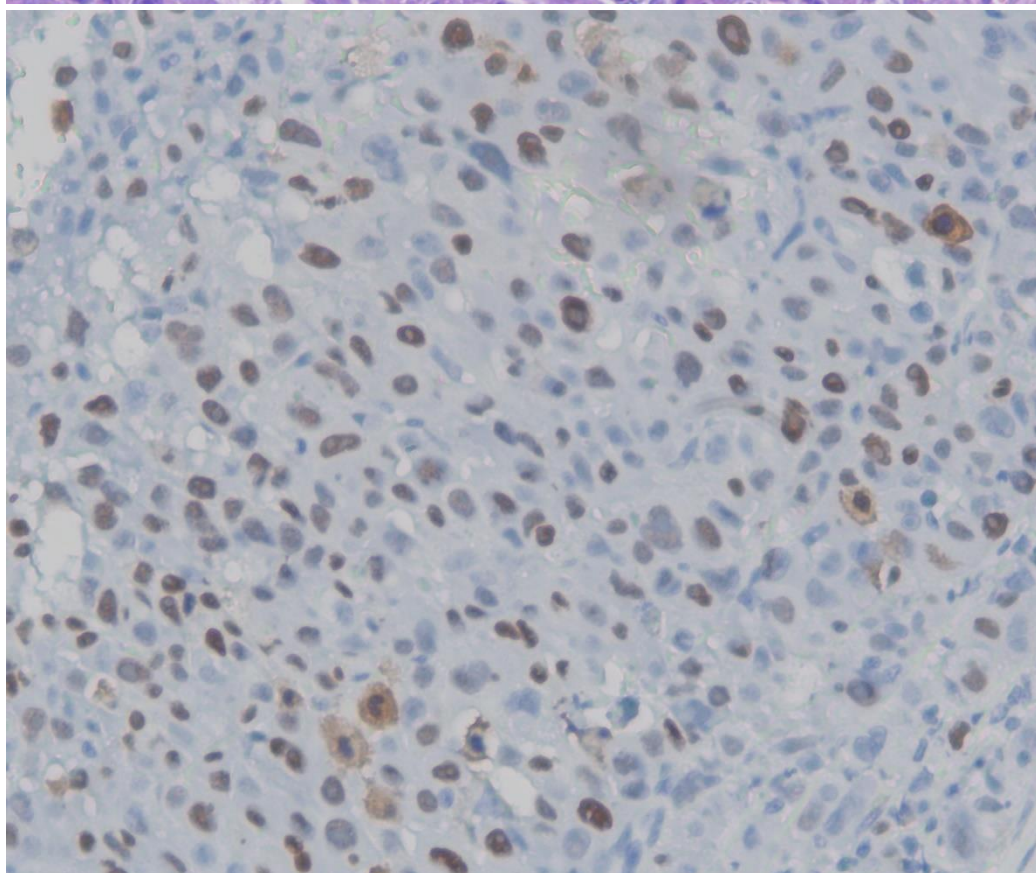

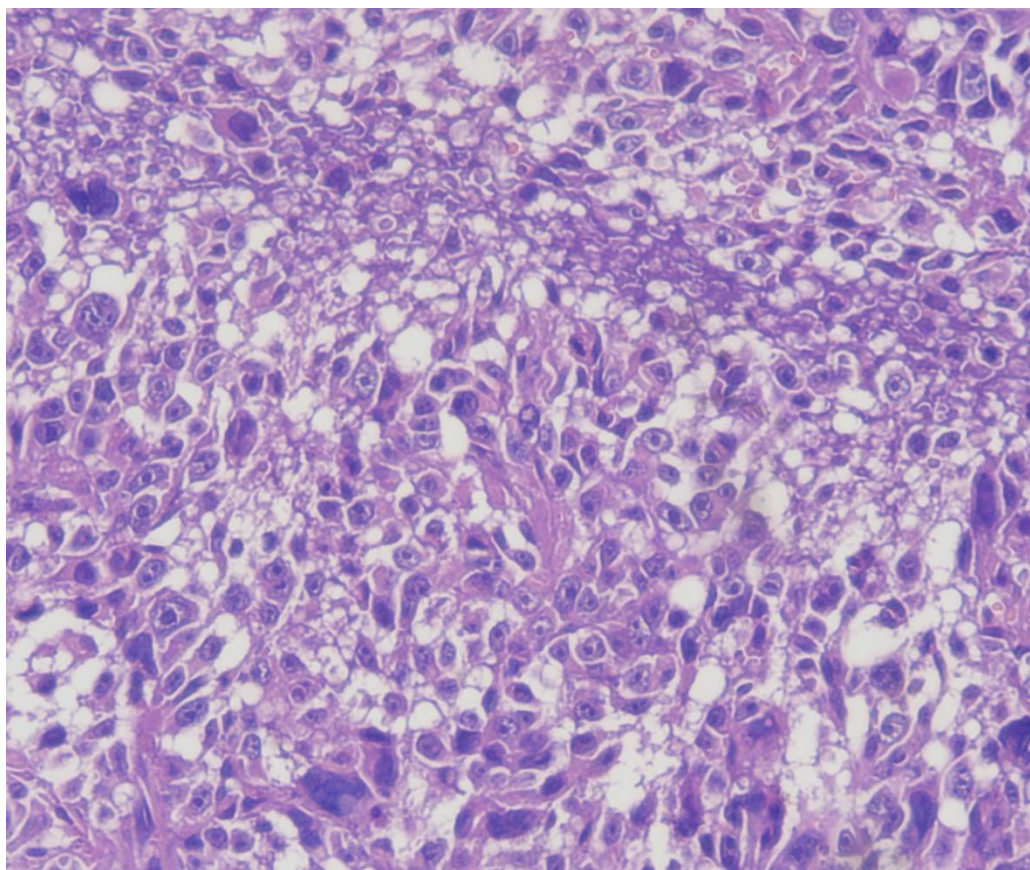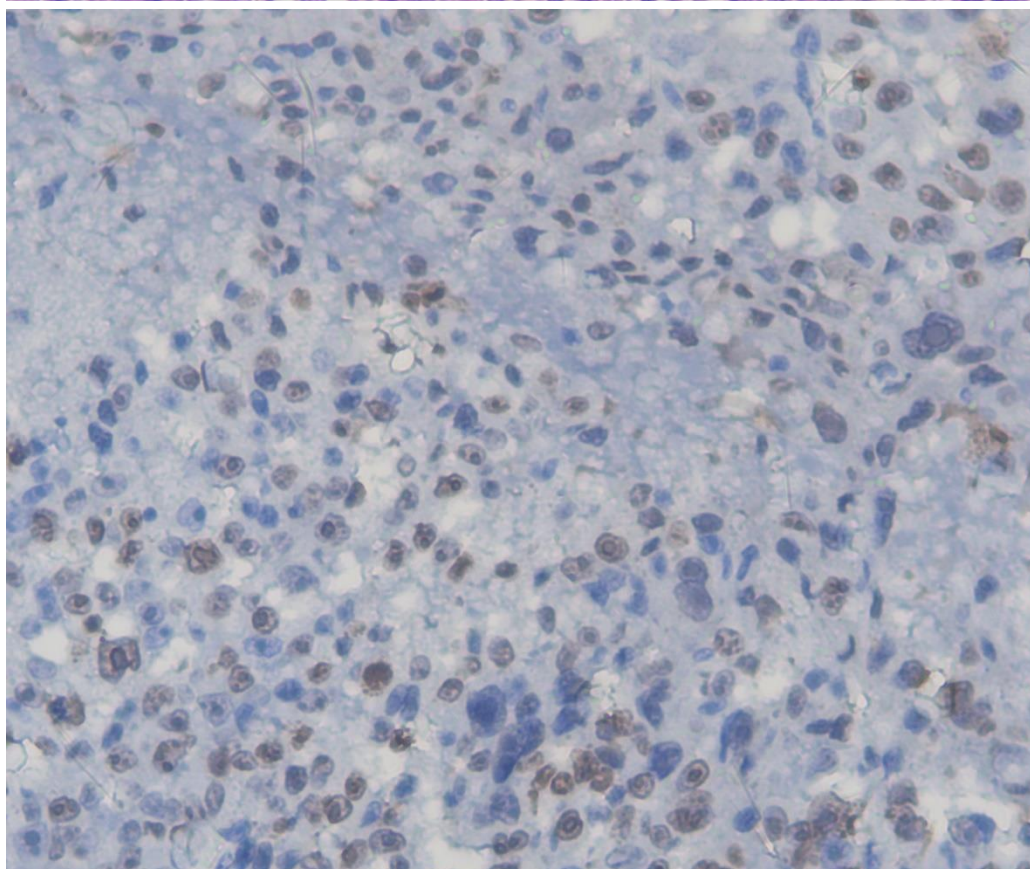

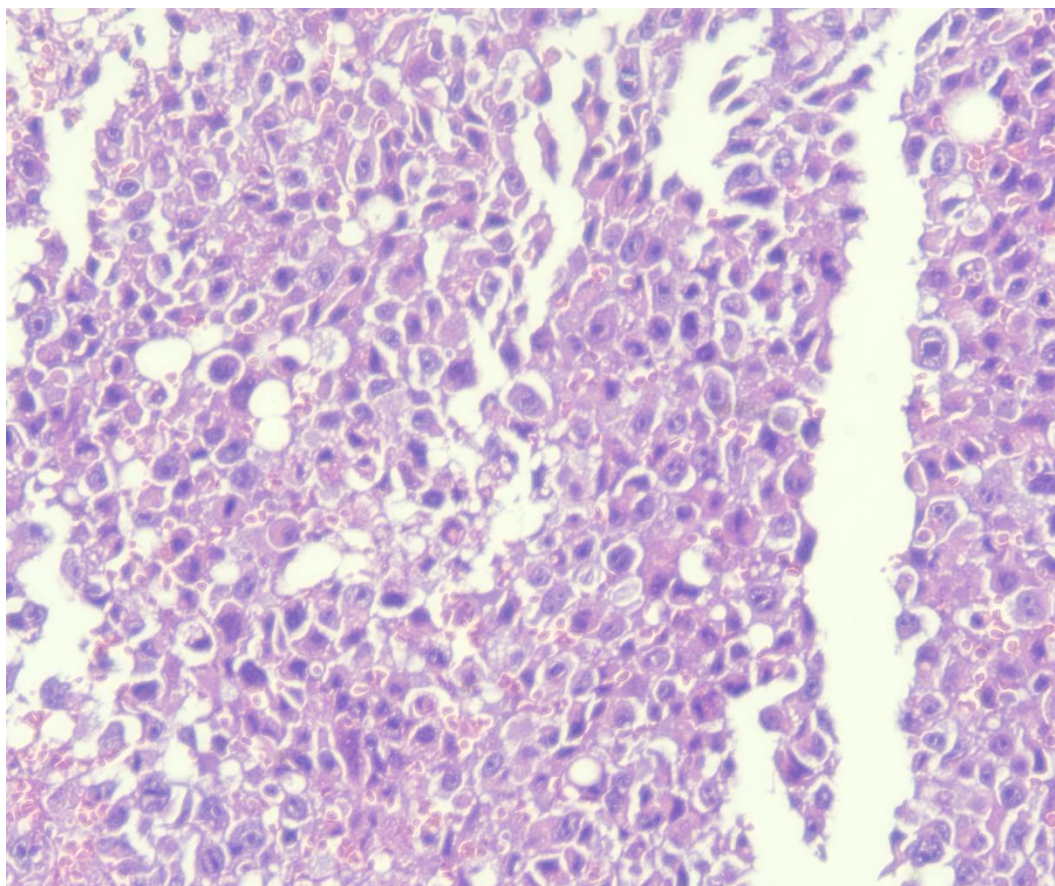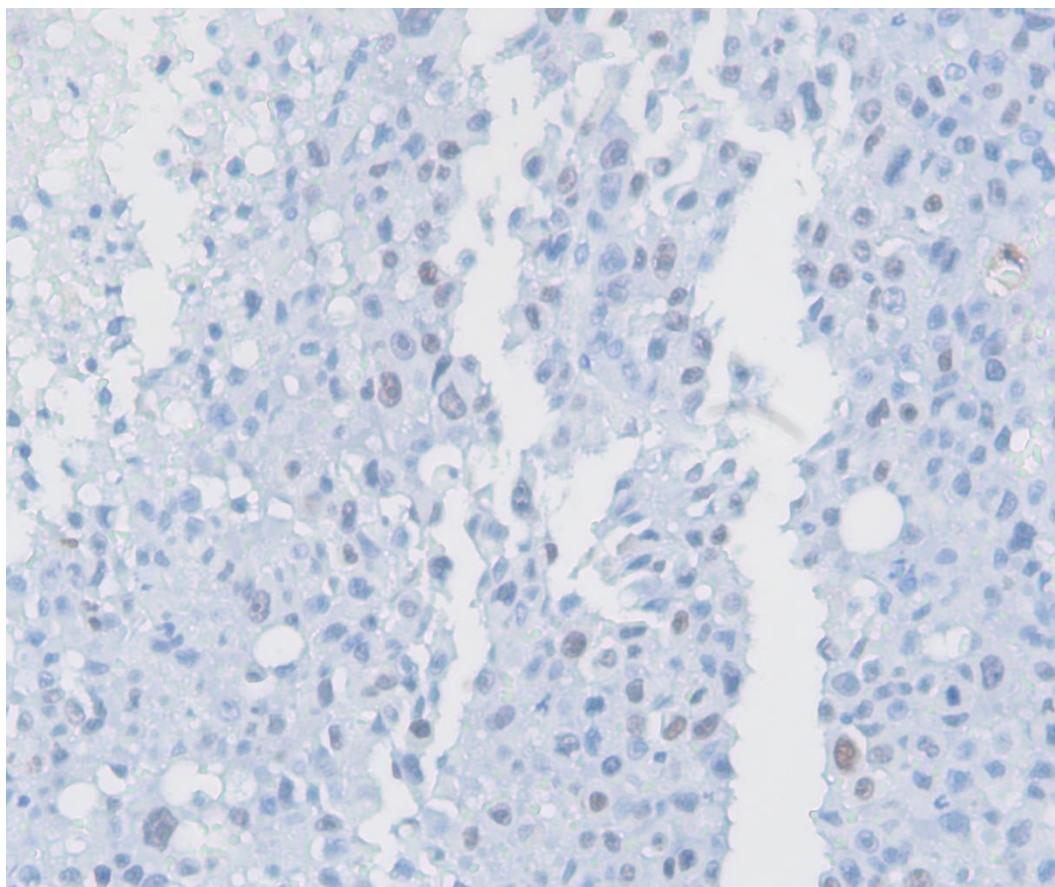

## KEGG Enrichment

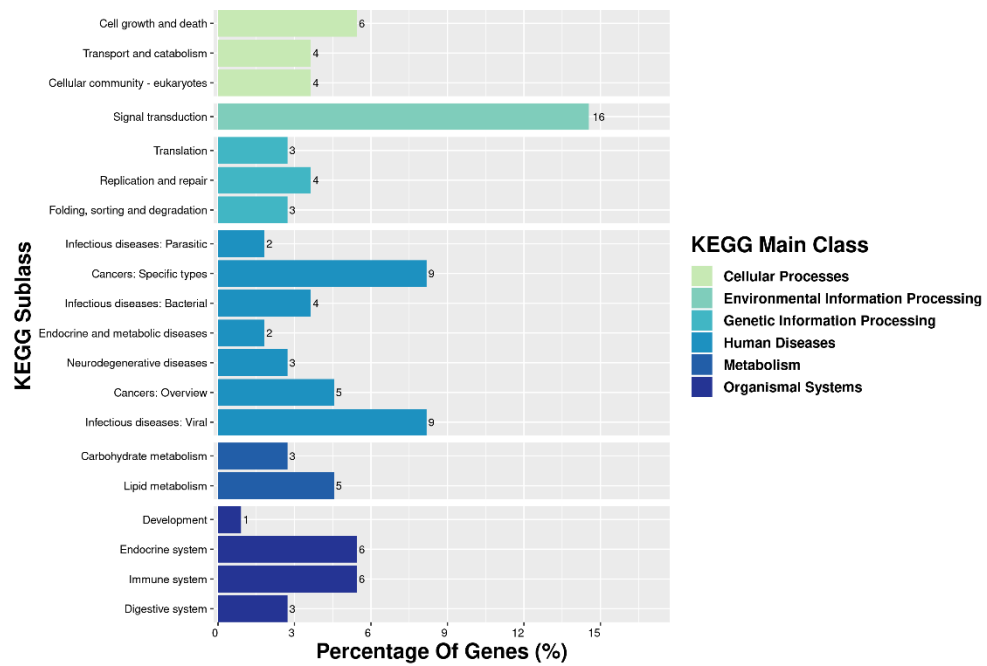

## Statistics of GO Enrichment

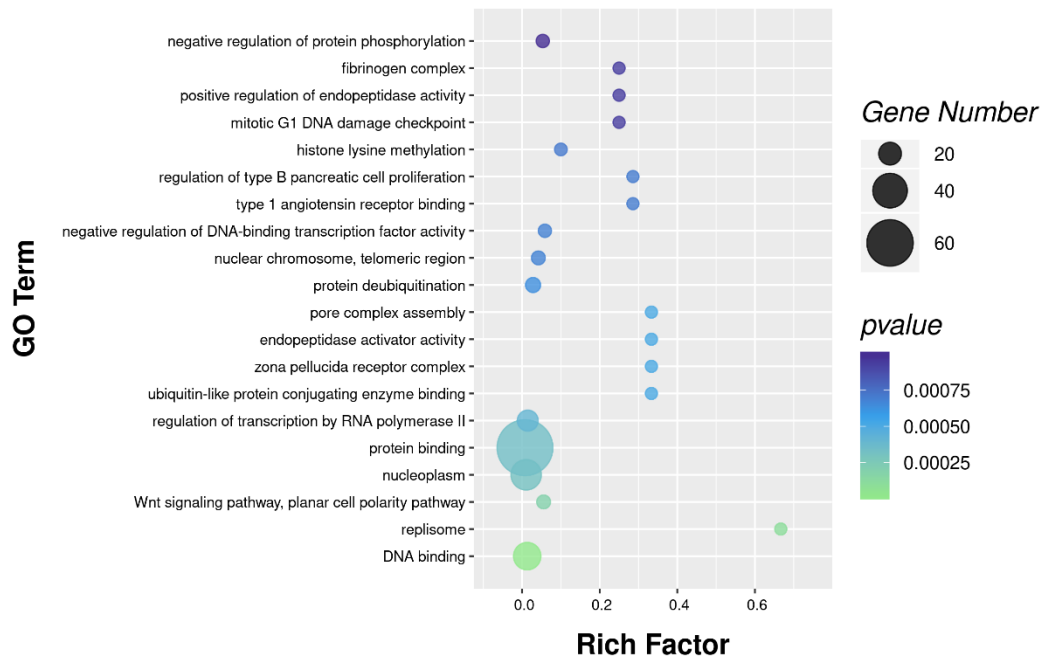

# Statistics of KEGG Enrichment

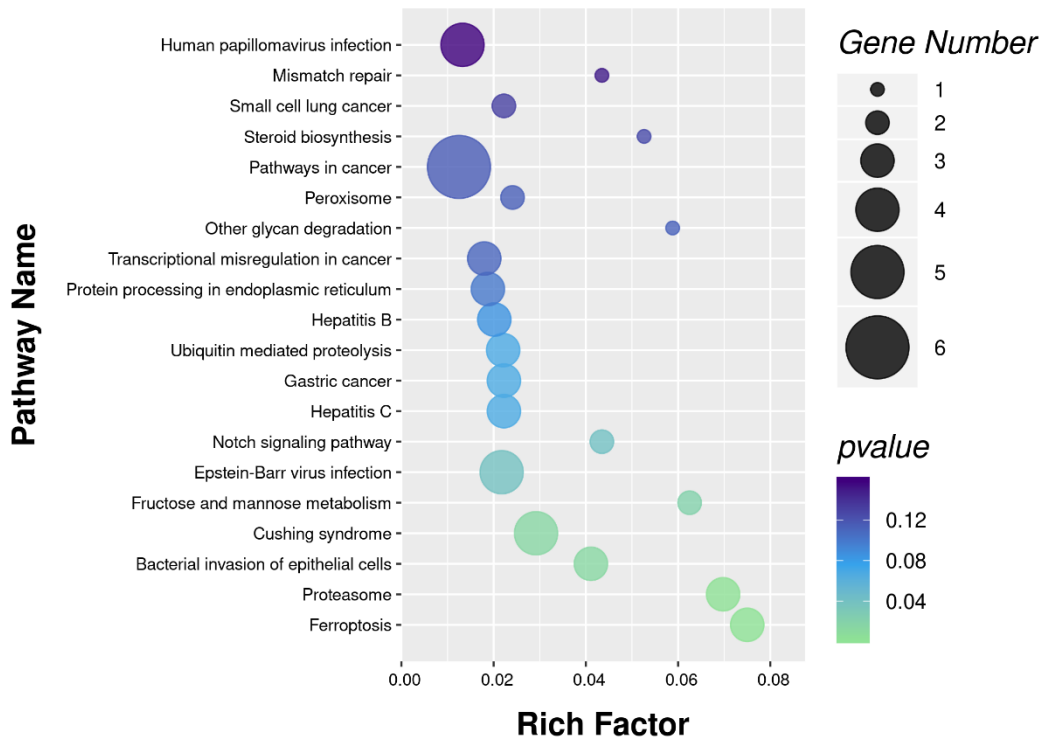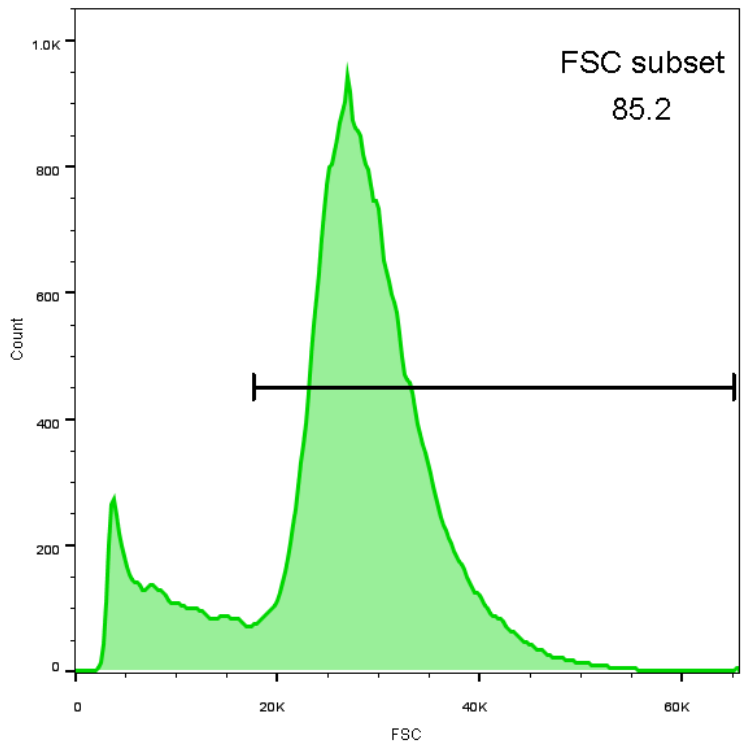

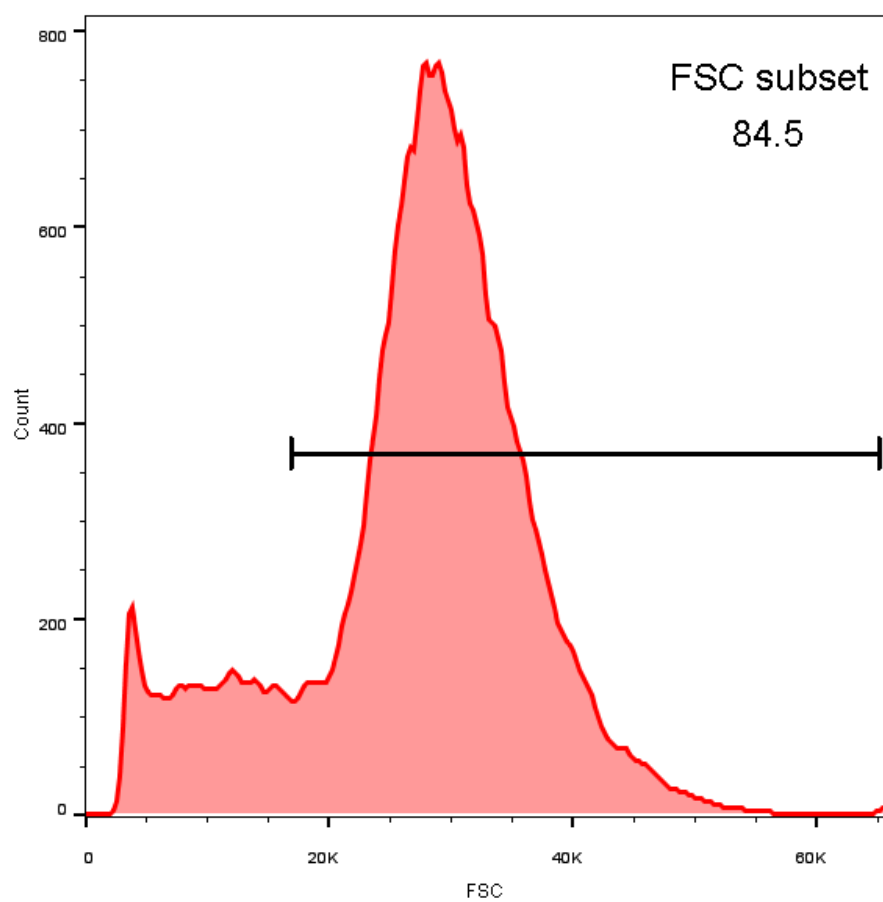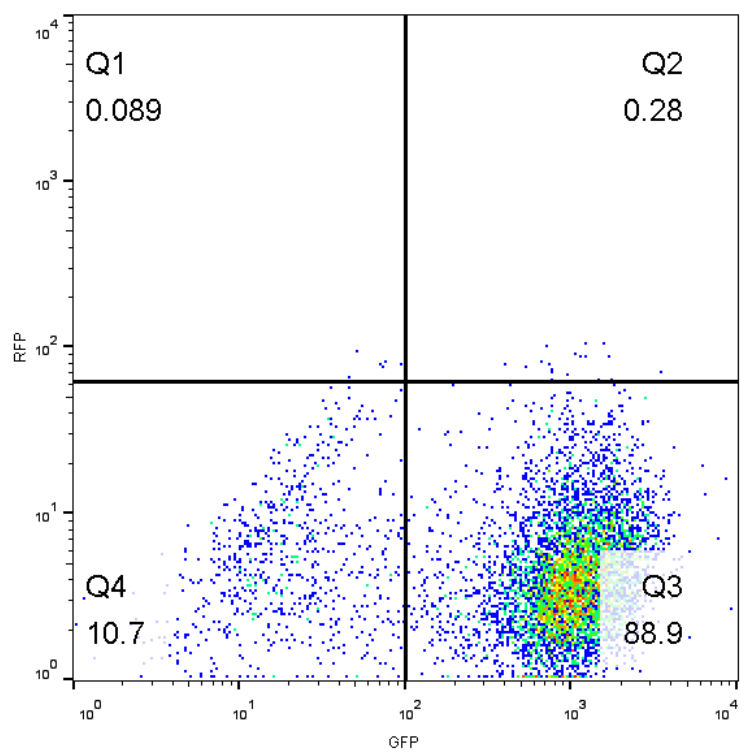

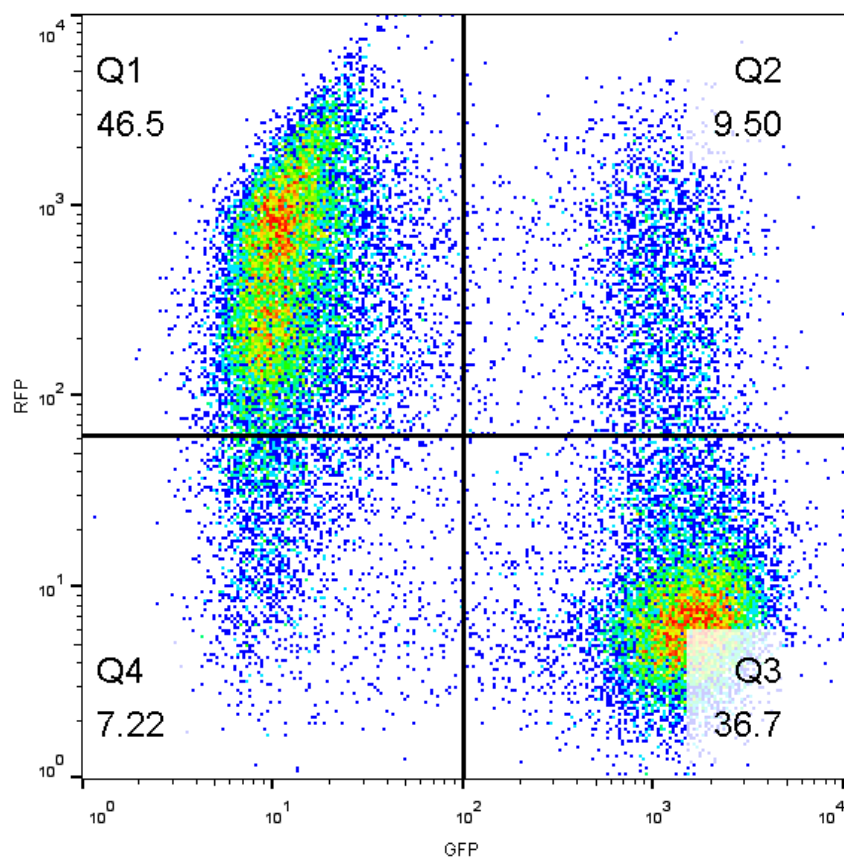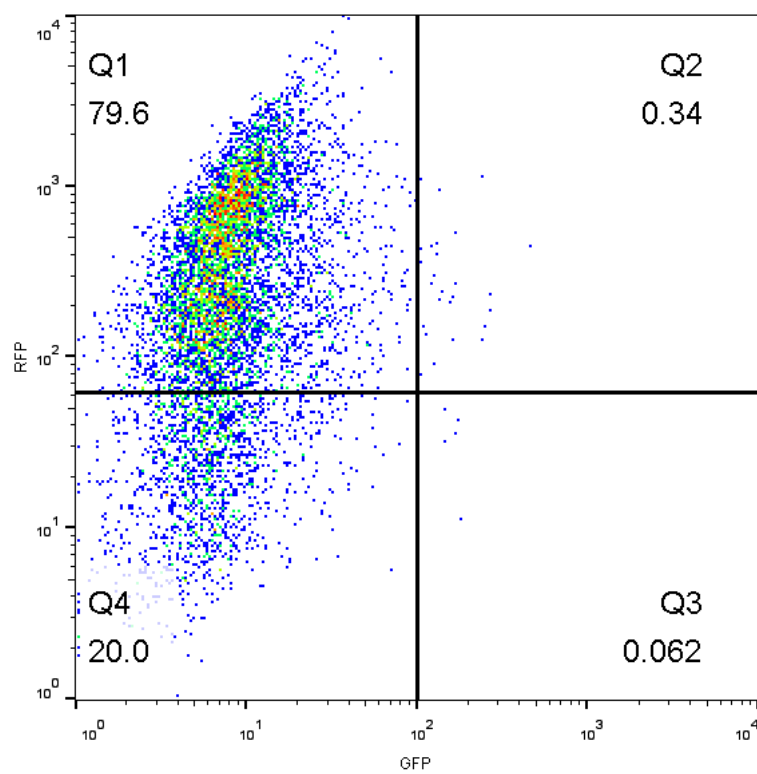

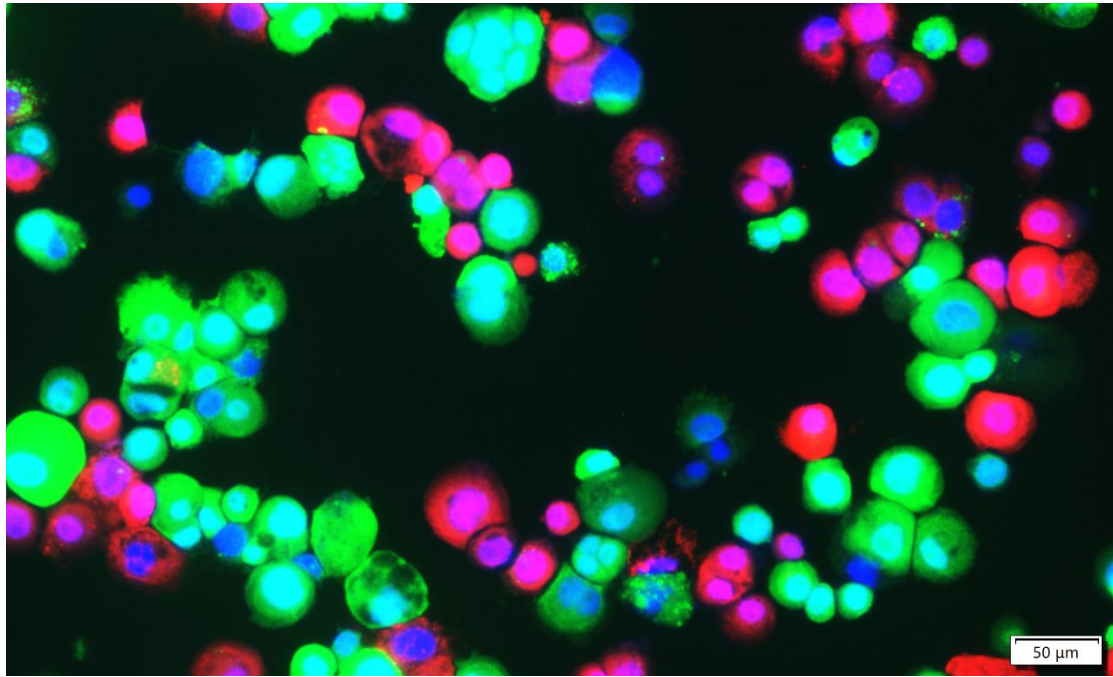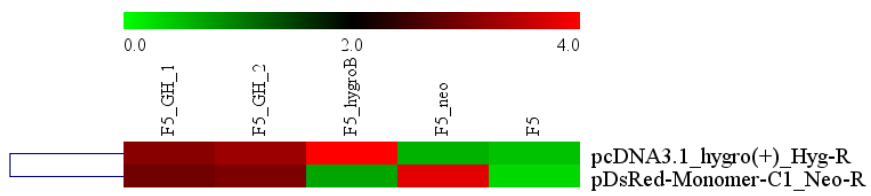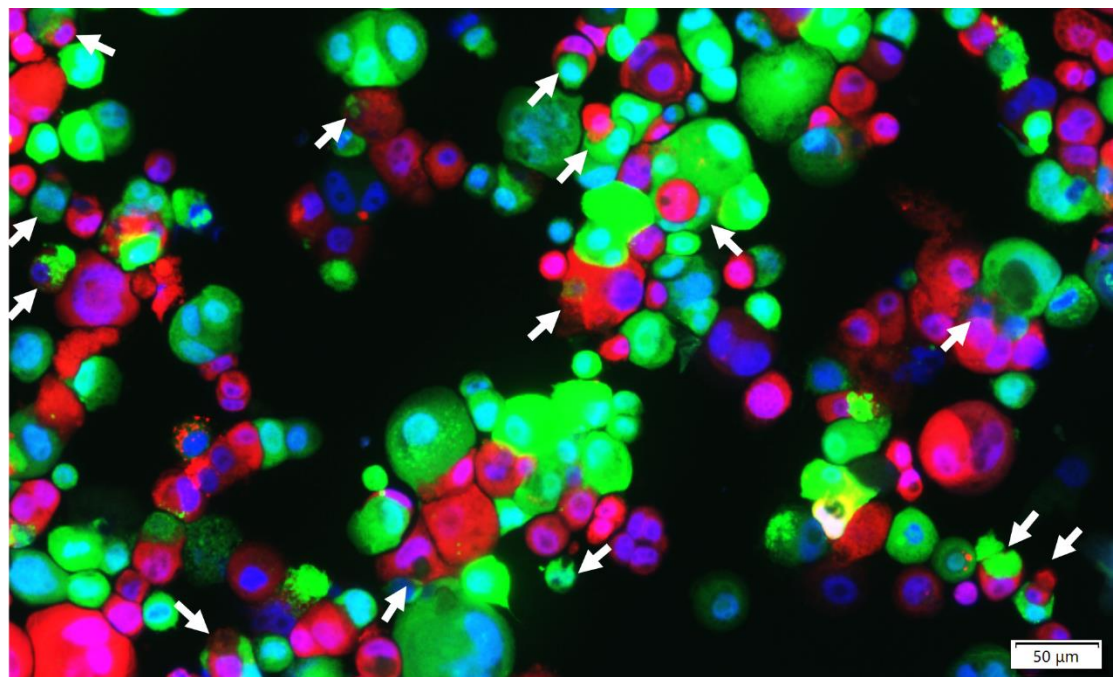

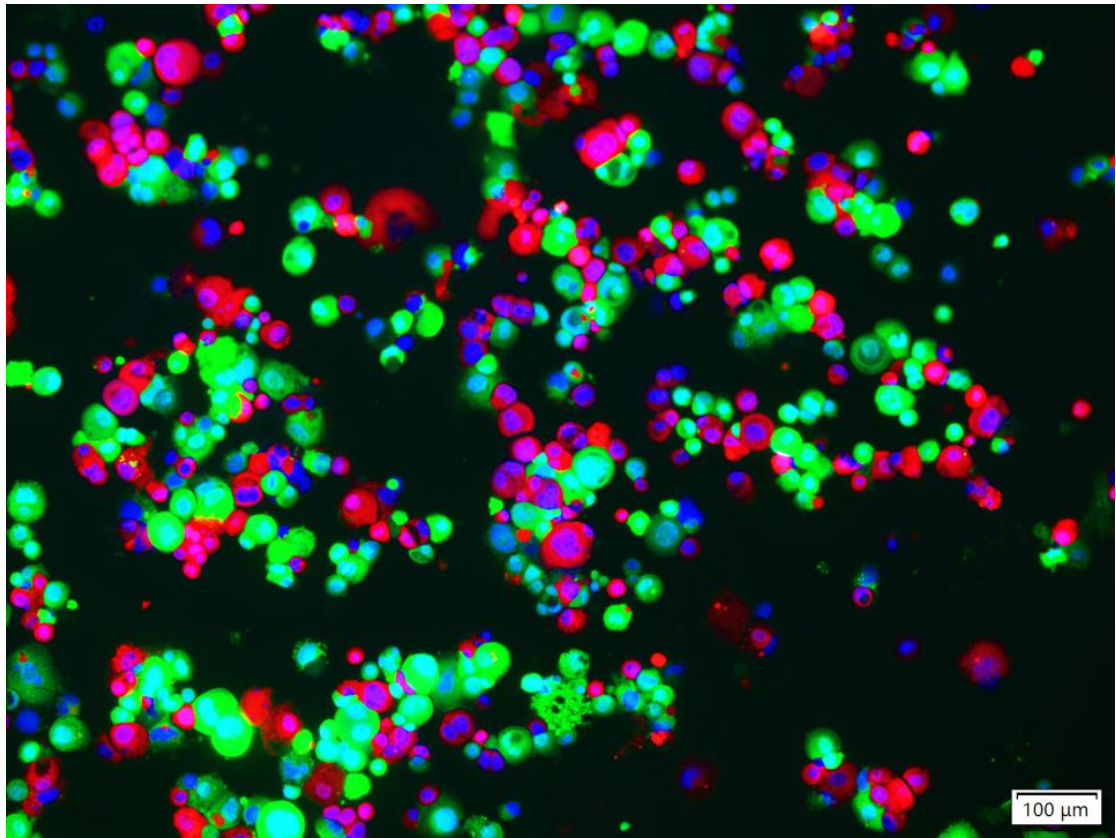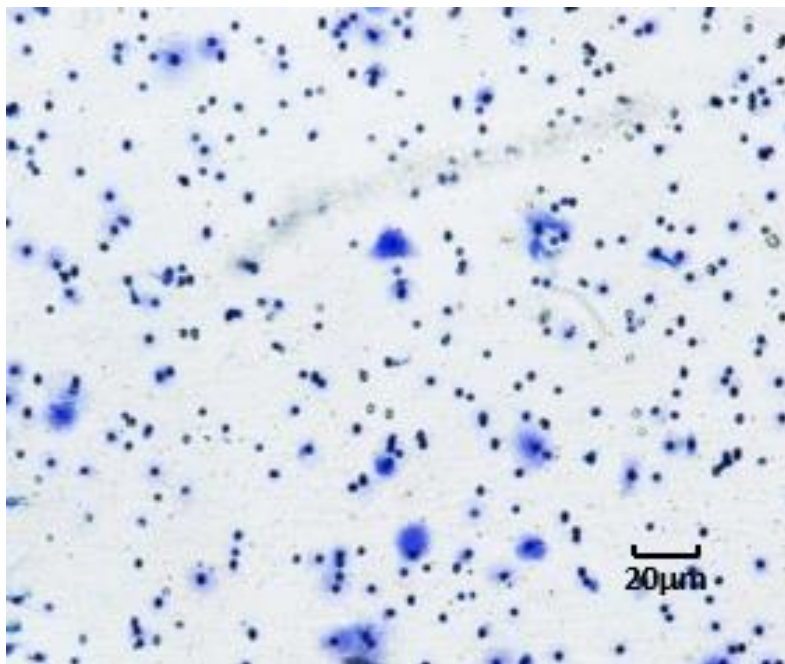

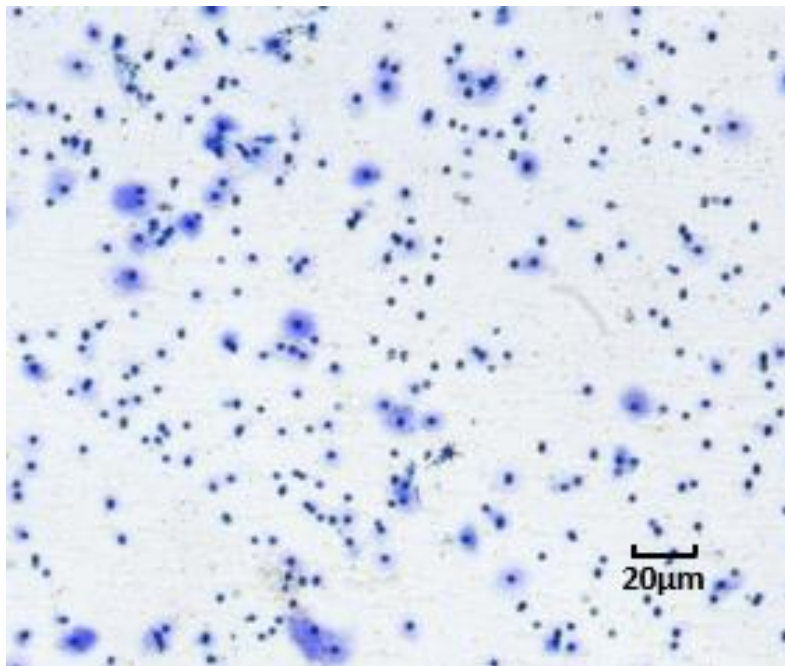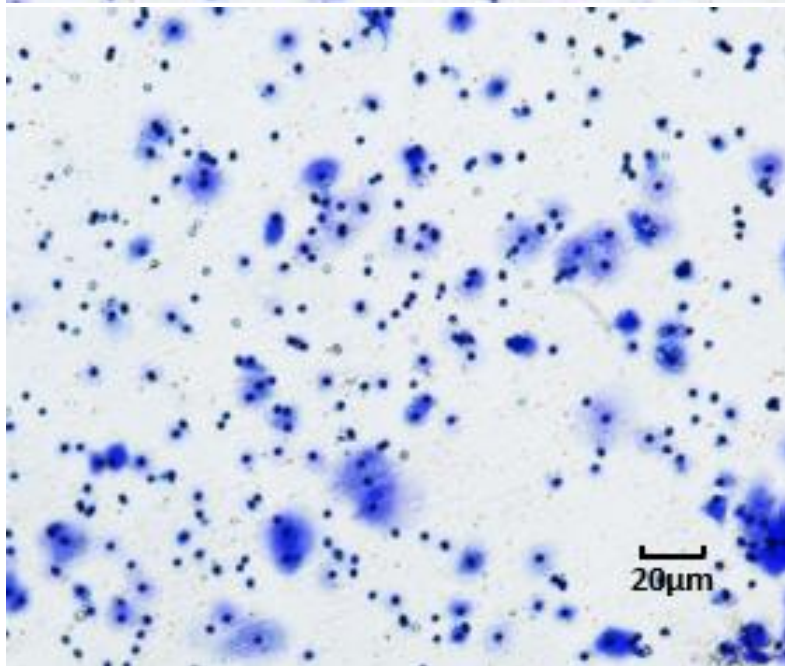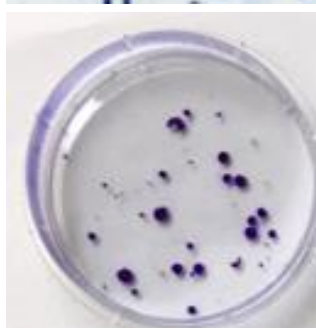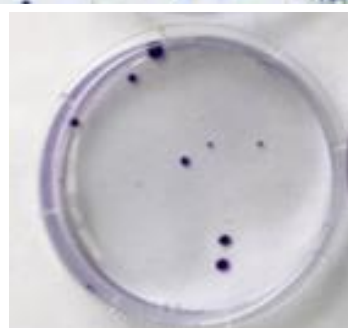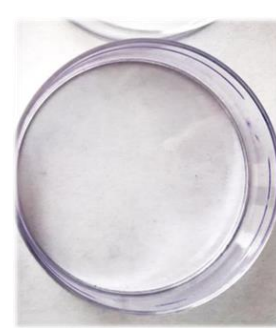

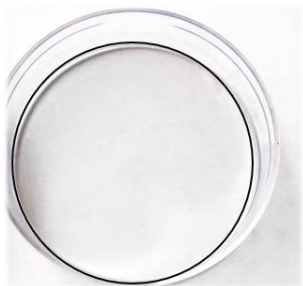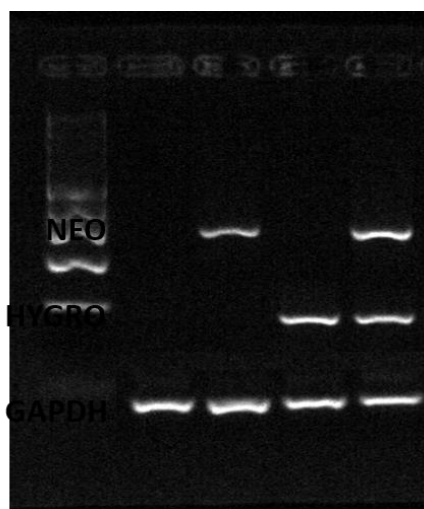

NEO

HYGRO

GAPDH

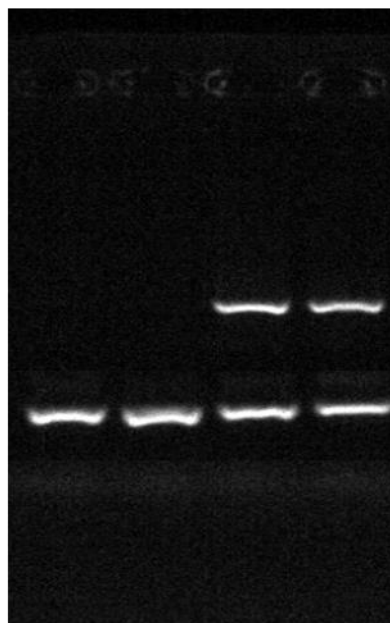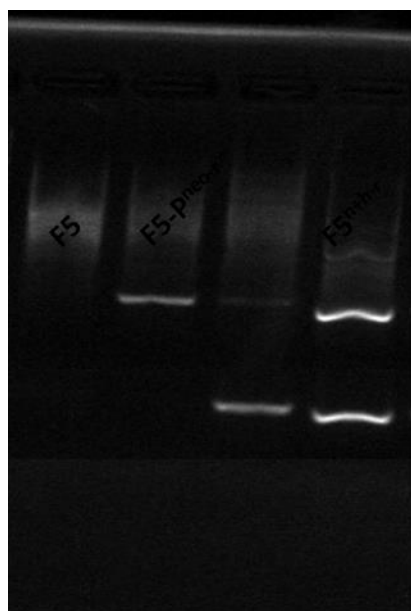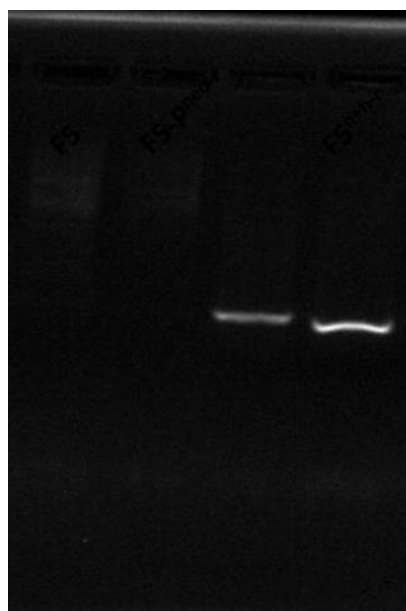

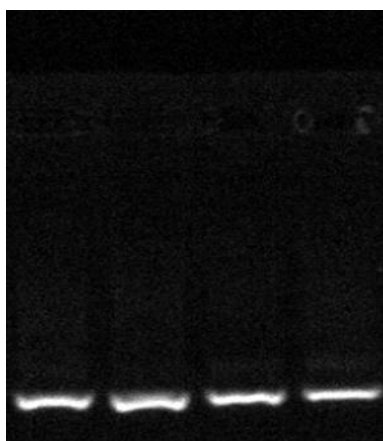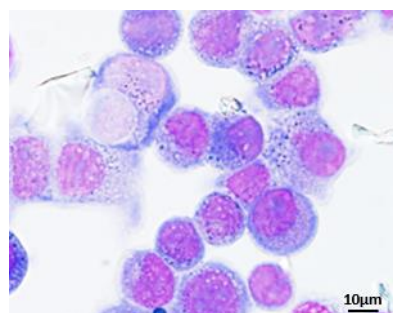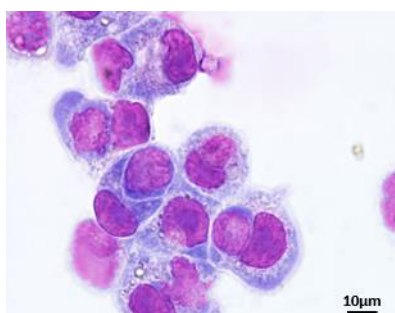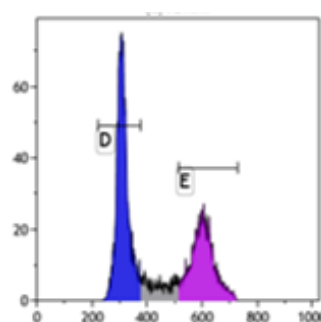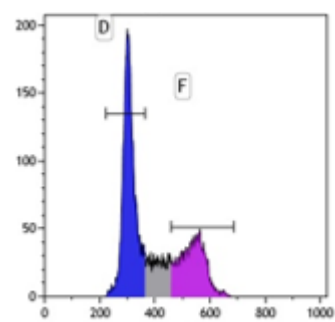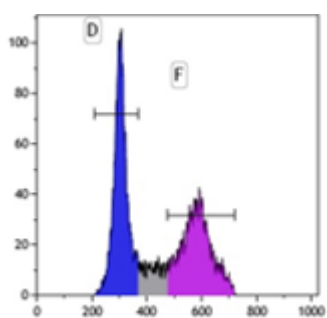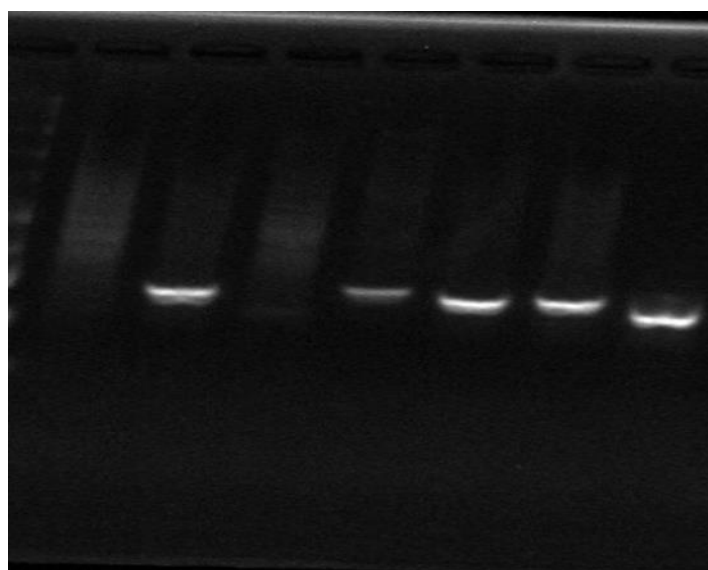

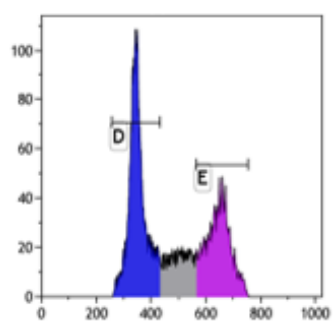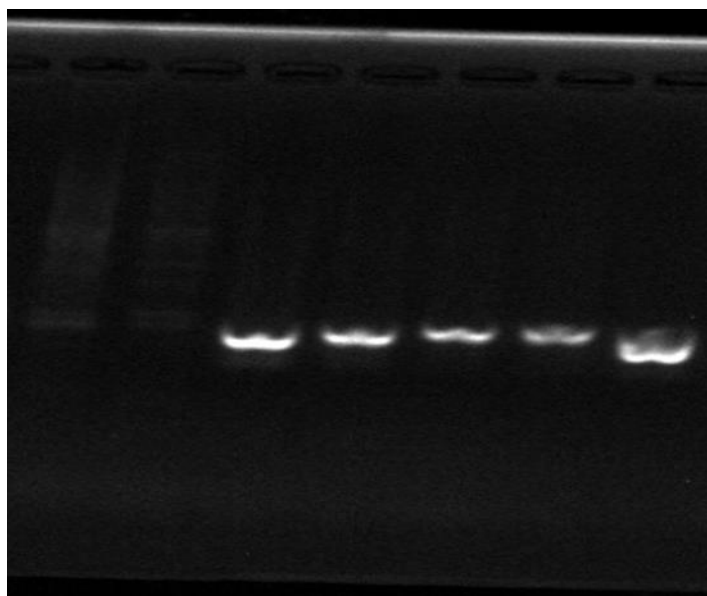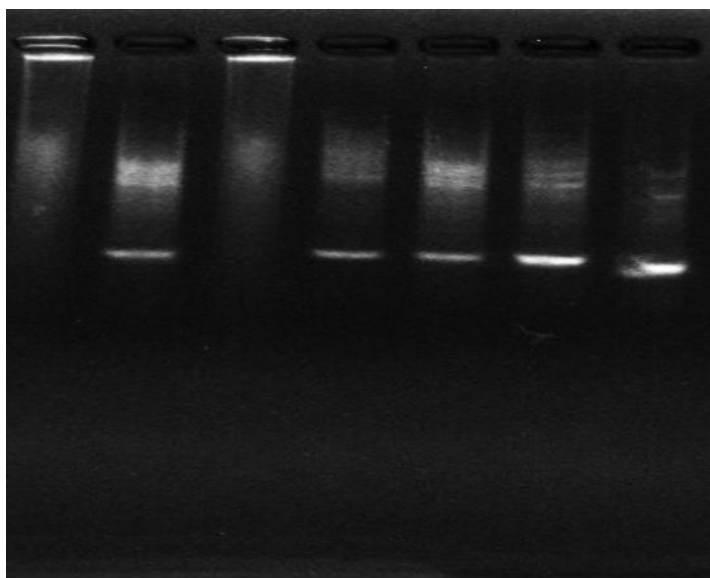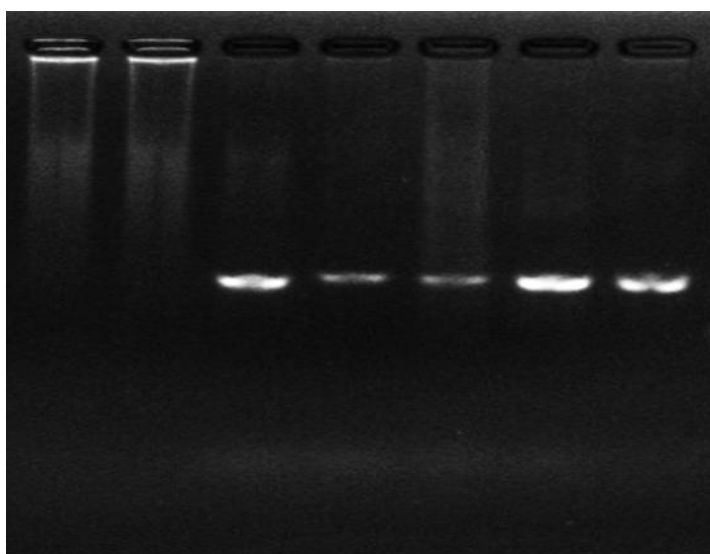

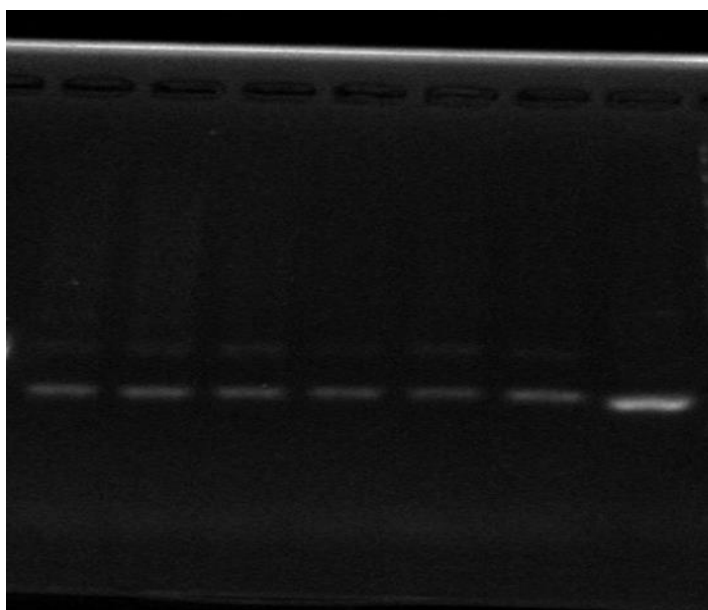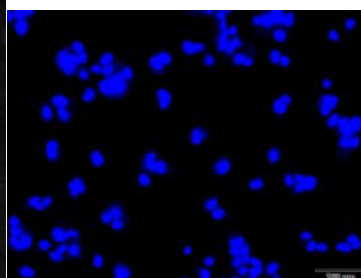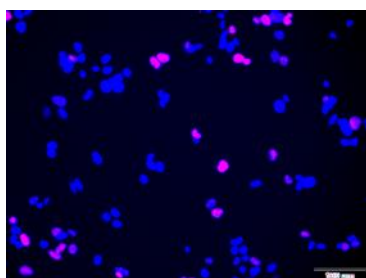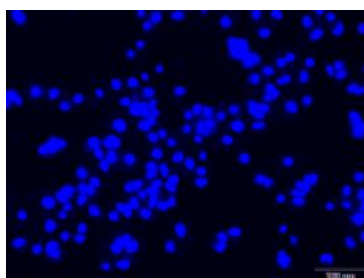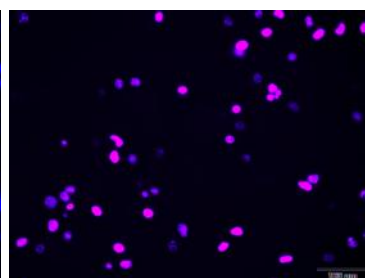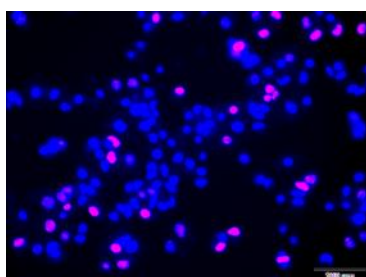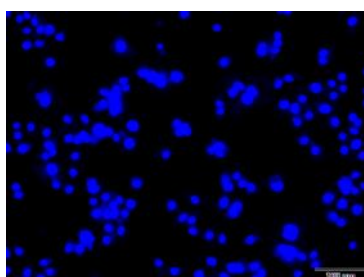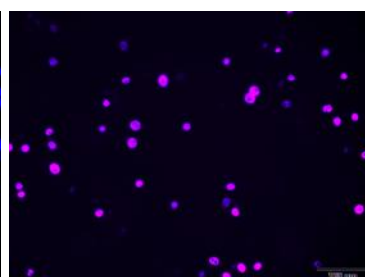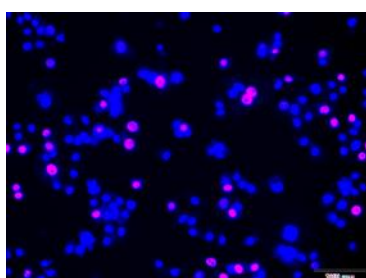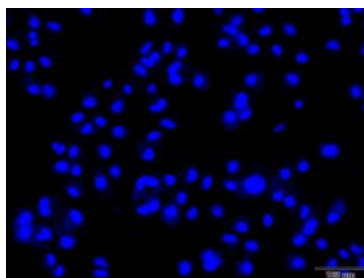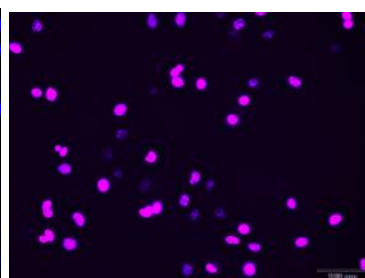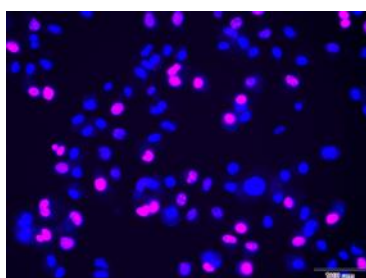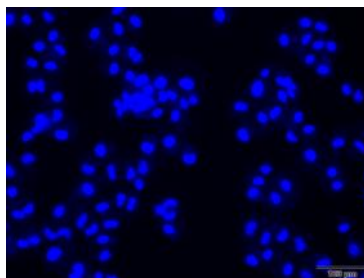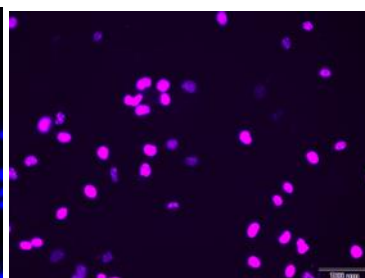

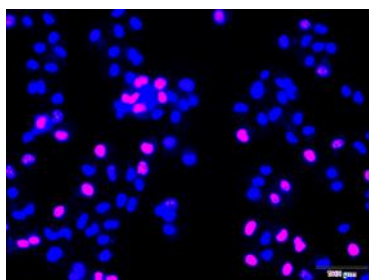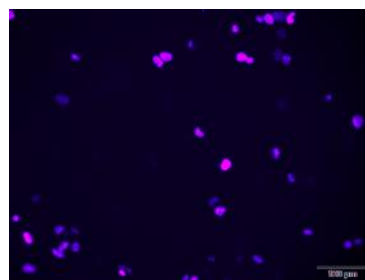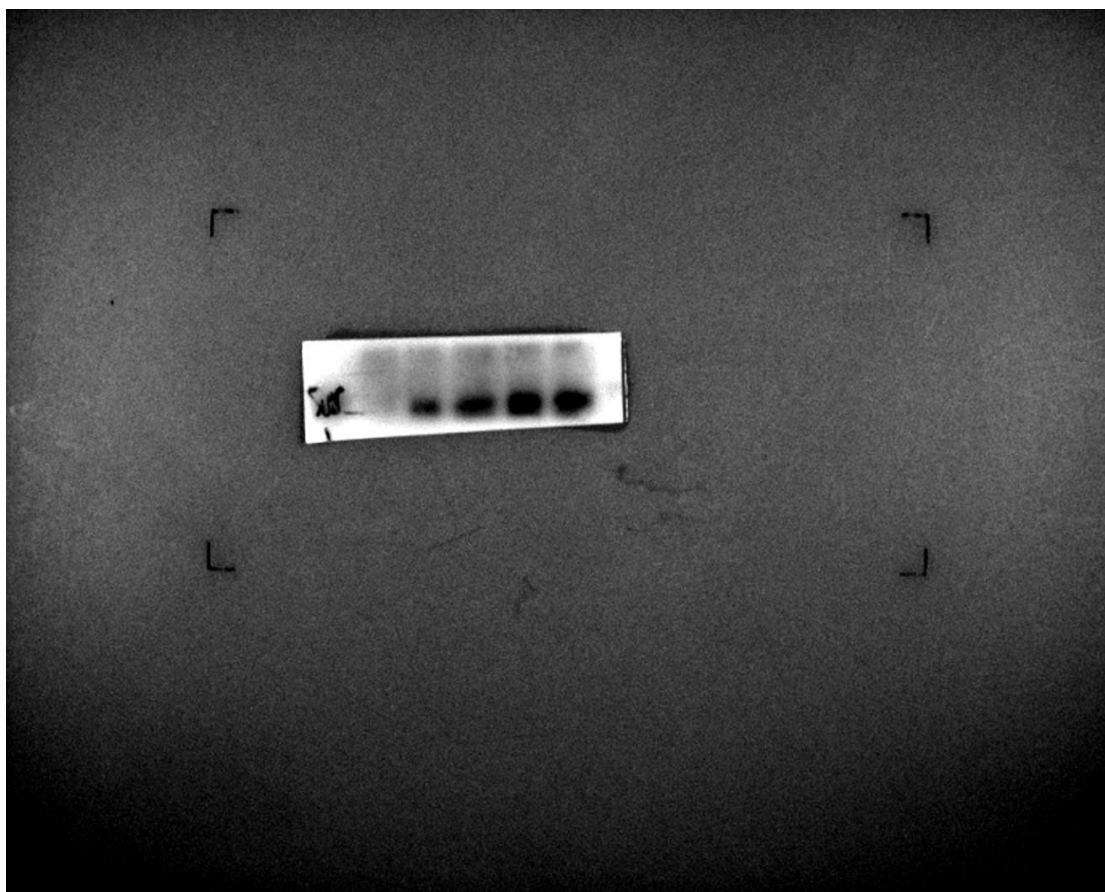

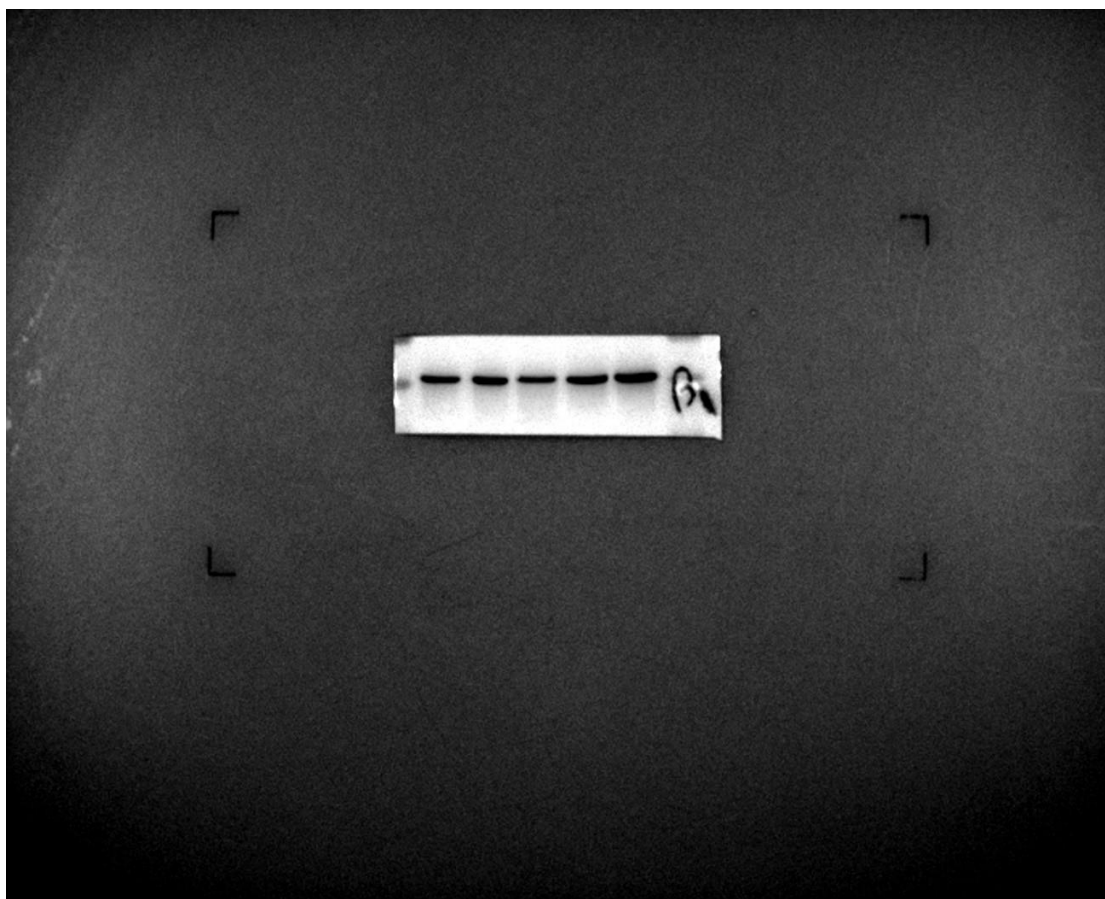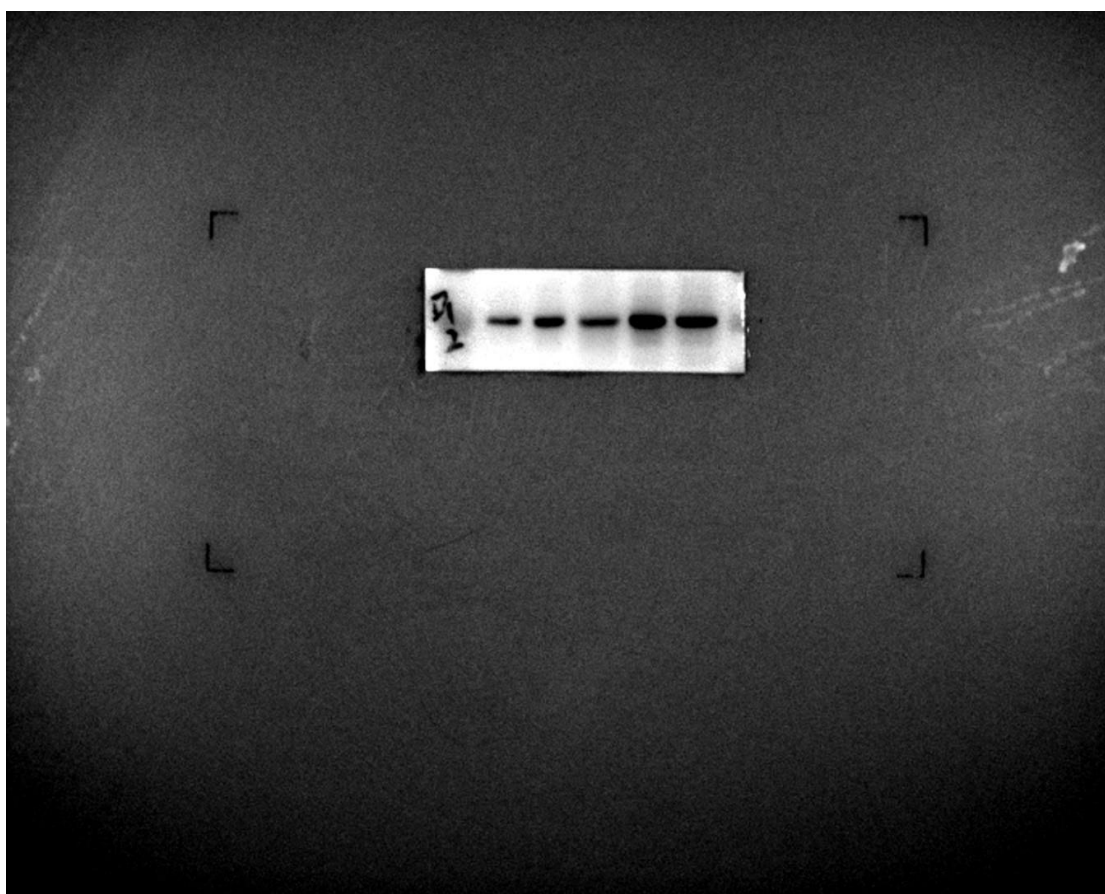

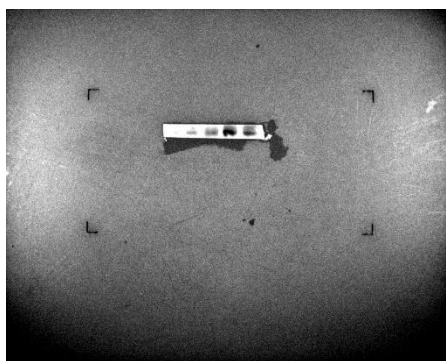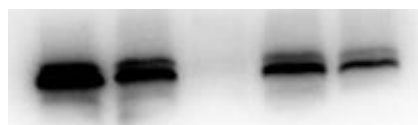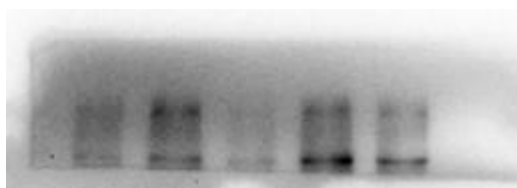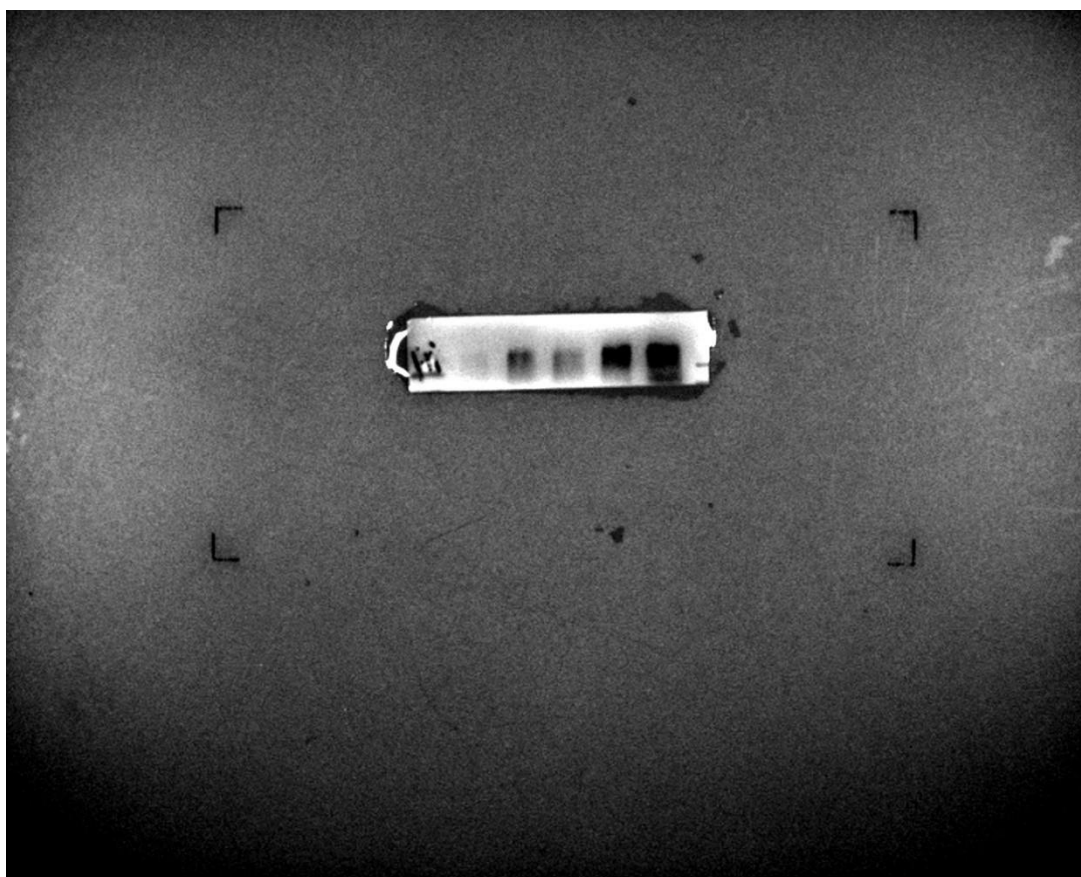

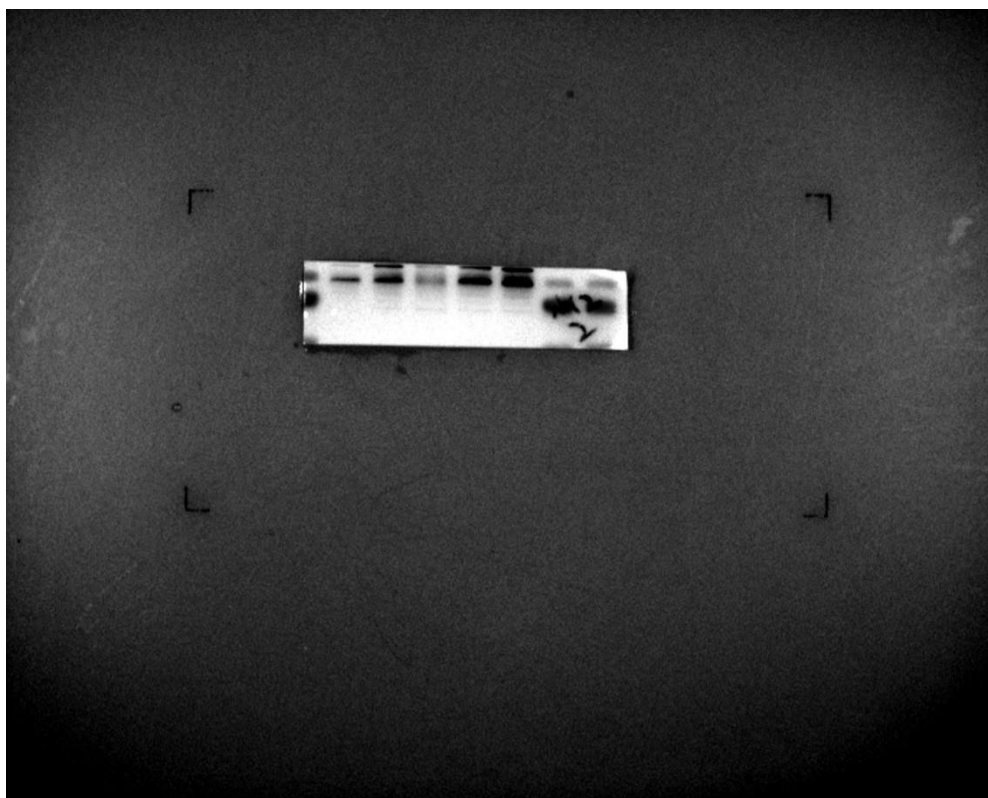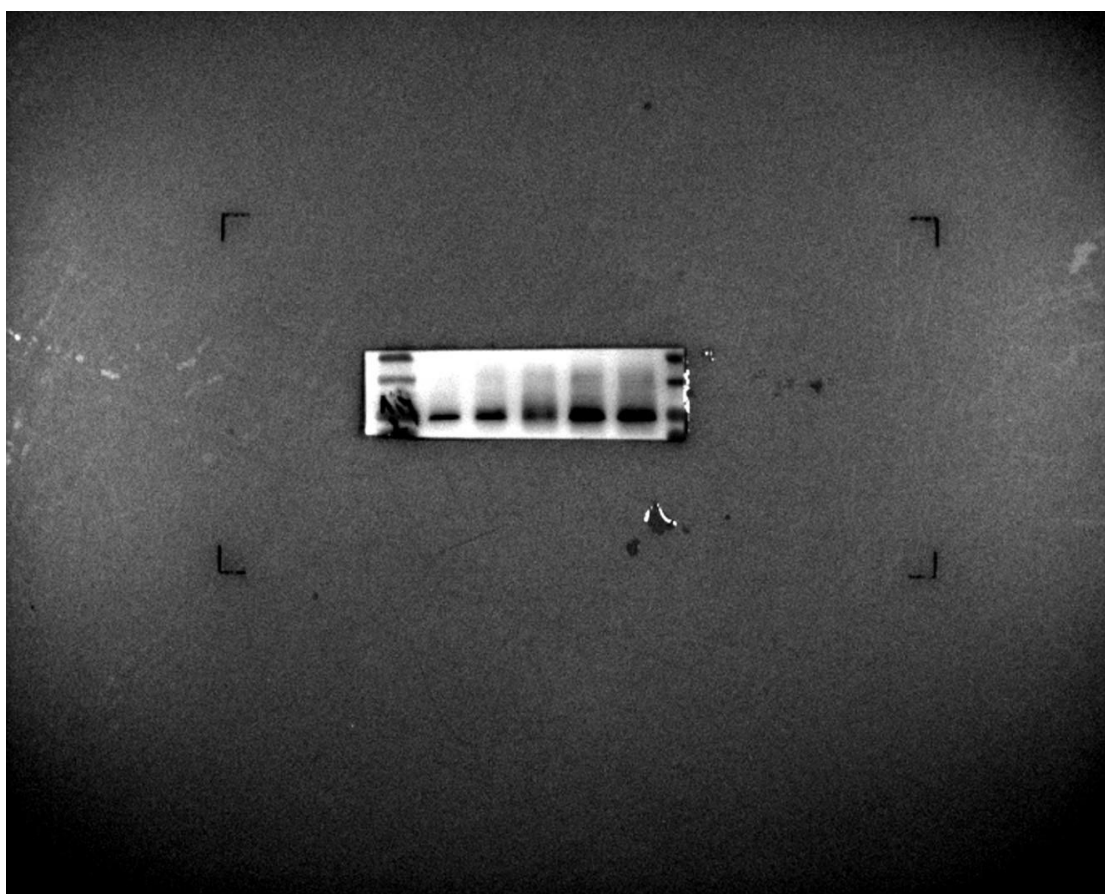

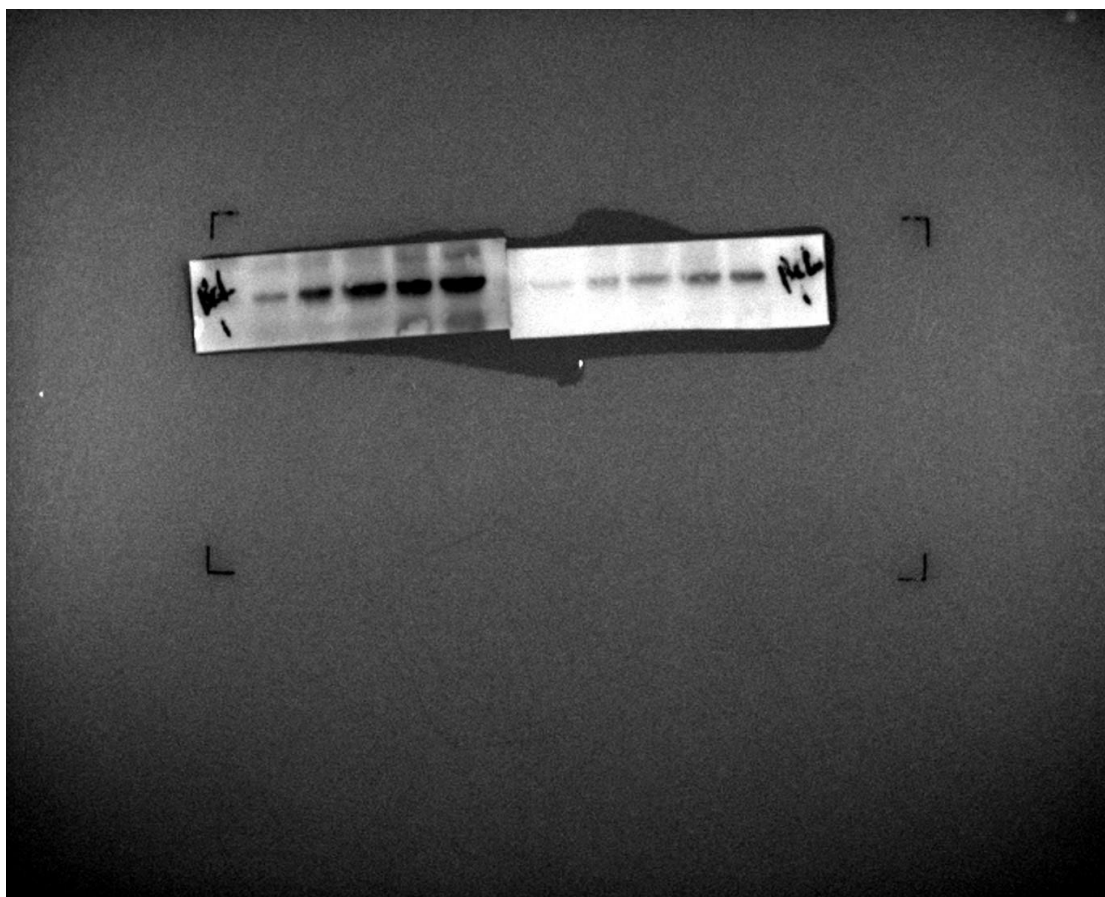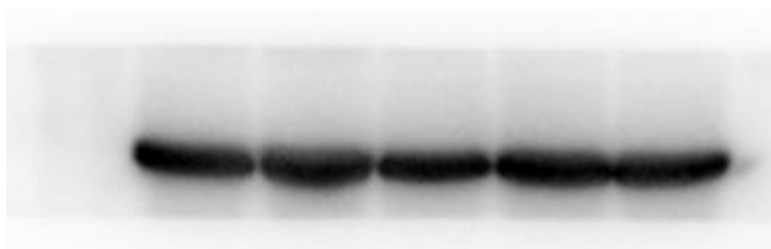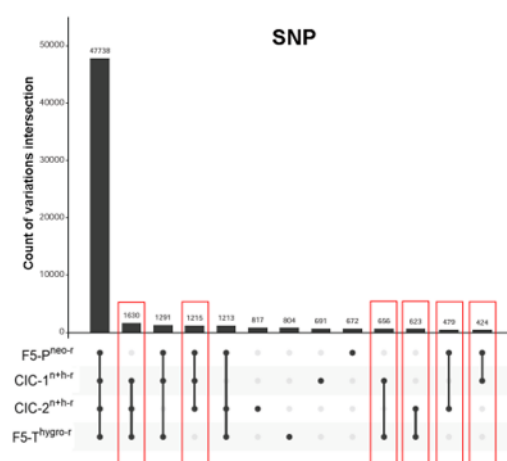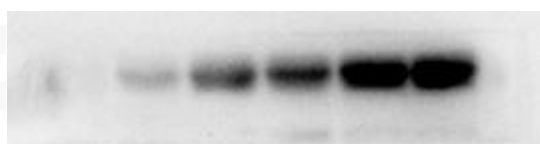

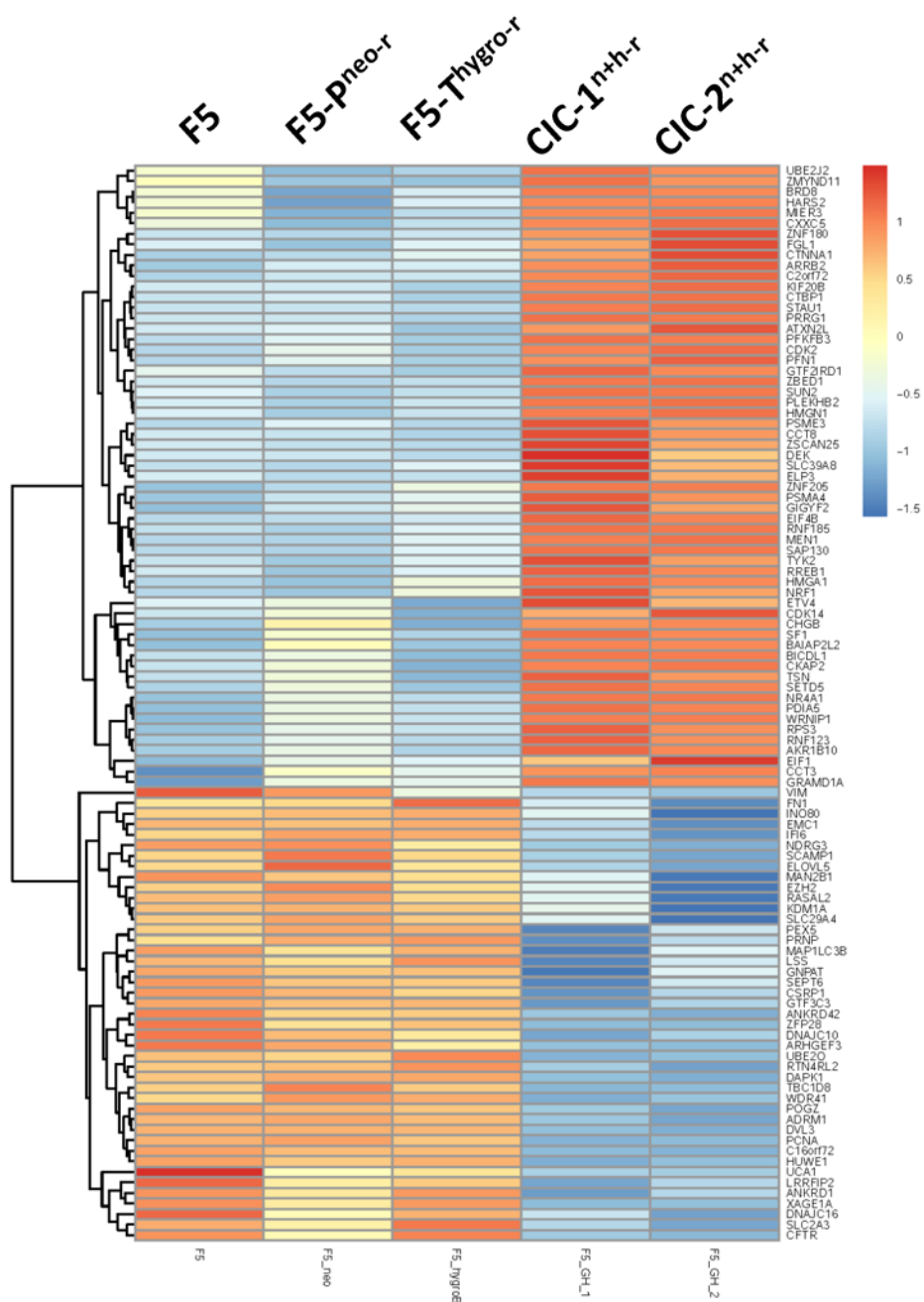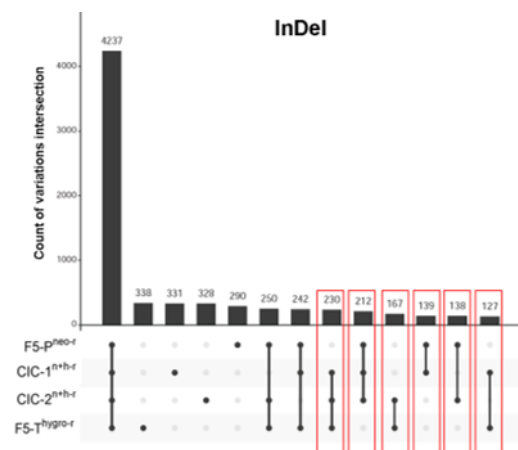

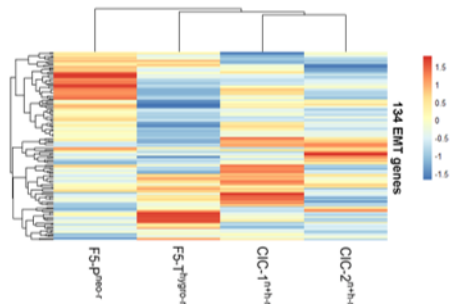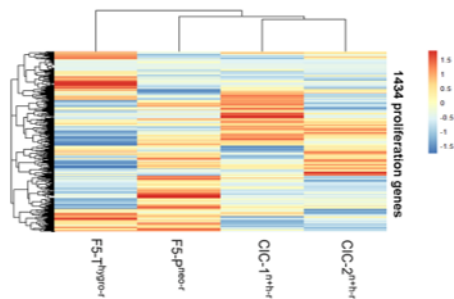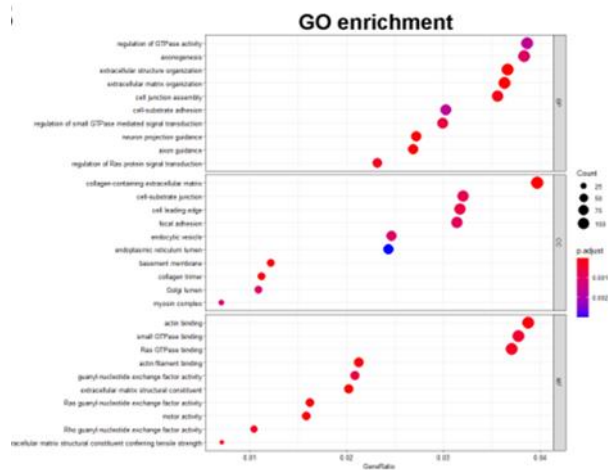

Supplement: Supplementary file 3 — Original Data File [file 41419_2023_5707_MOESM3_ESM.pdf]
