# Supplementary material for: Tumor malignancy by genetic transfer between cells forming cell-in-cell structures
Source: Cell Death Dis. 2023 Mar 13;14(3):195. doi: 10.1038/s41419-023-05707-1 (PMC10011543; doi:10.1038/s41419-023-05707-1)
Supplement: Supplementary file 5 — Table S1 [file 41419_2023_5707_MOESM5_ESM.docx]

Table S1 Primer sequences for RT-PCR gene expression studies

| Target | Sequence (5’-3’) |
| --- | --- |
| NEO | Forward: GGCTATTCGGCTATGACTGG  Reverse: TGGTCGAATGGGCAGGTA |
| HYGRO | Forward: TTCGGACCGCAAGGAA  Reverse: AGATGTTGGCGACCTCGTATT |
| CTBP1 | Forward: TCACAGGCCGGATCCCAGACAG  Reverse: GGTACCTATAGGCAGCCCCATTGAGC |
| CDK2 | Forward: GTACCTCCCCTGGATGAAGAT  Reverse: CGAAATCCGCTTGTTAGGGTC |
| HMGA1 | Forward: AAGGGGCAGACCCAAAAA  Reverse: TCCAGTCCCAGAAGGAAGC |
| CKAP2 | Forward: GCAAGATGCTAACATGCCCAA  Reverse: TGGCTTTAGGTATAGTGGCTGA |
| AKR1B10 | Forward: CCCAGGTTCTGATCCGTTTC  Reverse: GGTTGCCATCTCCTCATCAC |
| DAPK-1 | Forward: GAATCCTAGACGTGGTCCGGTAT  Reverse: CCTCGGTGCGTATCCTTTCG |
| RASAL2 | Forward: AGCAGAAAGGTCCCCTCGTAG  Reverse: AGGGTGAGGTATTTGCAGTGT |
| HUWE1 | Forward: CAAGTAGCCATCAGCAAGA  Reverse: GTCCTCCAGTTCATTCTCAA |
| DNAJC10 | Forward: CACAGACACGACTCAGGCTT  Reverse: GGTTATCCTGGGTGGCACAG |
| CFTR | Forward: GTGTGATTCCACCTTCTCCAA  Reverse: GCCTGGCACCATTAAAGAAA |
| GAPDH | Forward: TCCCATCACCATCTTCCAG  Reverse: TCCACCACTGACACGTTG |
