# Supplementary material for: Tumor malignancy by genetic transfer between cells forming cell-in-cell structures
Source: Cell Death Dis. 2023 Mar 13;14(3):195. doi: 10.1038/s41419-023-05707-1 (PMC10011543; doi:10.1038/s41419-023-05707-1)
Supplement: Supplementary file 6 — Table S2 [file 41419_2023_5707_MOESM6_ESM.docx]

Table S2 List of Genes altered by CICs process in PLC/PRF/5 cells.

| **Gene name** | **F5** | **F5-P^neo-r^** | **F5-T^hygro-r^** | **CIC-1^n+h-r^** | **CIC-2^n+h-r^** |
| --- | --- | --- | --- | --- | --- |
| *UBE2J2* | -0.17 | -1.05 | -0.85 | 1.12 | 0.95 |
| *ZMYND11* | -0.04 | -0.96 | -1.01 | 1.1 | 0.92 |
| *BRD8* | -0.21 | -1.23 | -0.6 | 1.05 | 0.99 |
| *HARS2* | -0.21 | -1.25 | -0.58 | 1 | 1.03 |
| *MIER3* | -0.19 | -1.12 | -0.76 | 1 | 1.07 |
| *CXXC5* | -0.31 | -1.08 | -0.72 | 0.97 | 1.13 |
| *ZNF180* | -0.69 | -0.82 | -0.66 | 0.88 | 1.28 |
| *FGL1* | -0.59 | -0.99 | -0.53 | 0.81 | 1.31 |
| *CTNNA1* | -0.88 | -0.78 | -0.47 | 0.83 | 1.31 |
| *ARRB2* | -0.94 | -0.61 | -0.61 | 0.94 | 1.22 |
| *C2orf72* | -0.85 | -0.66 | -0.67 | 1 | 1.18 |
| *KIF20B* | -0.65 | -0.65 | -0.88 | 1.03 | 1.15 |
| *CTBP1* | -0.69 | -0.6 | -0.89 | 1.08 | 1.1 |
| *STAU1* | -0.7 | -0.69 | -0.79 | 1.05 | 1.14 |
| *PRRG1* | -0.66 | -0.68 | -0.84 | 1.12 | 1.07 |
| *ATXN2L* | -0.64 | -0.53 | -0.97 | 0.9 | 1.25 |
| *PFKFB3* | -0.76 | -0.51 | -0.9 | 1.11 | 1.06 |
| *CDK2* | -0.81 | -0.42 | -0.91 | 1.02 | 1.13 |
| *PFN1* | -0.81 | -0.48 | -0.87 | 0.96 | 1.2 |
| *GTF2IRD1* | -0.46 | -0.77 | -0.92 | 1.18 | 0.97 |
| *ZBED1* | -0.63 | -0.81 | -0.74 | 1.07 | 1.11 |
| *SUN2* | -0.55 | -0.89 | -0.73 | 1.1 | 1.07 |
| *PLEKHB2* | -0.62 | -0.88 | -0.68 | 1.08 | 1.1 |
| *HMGN1* | -0.58 | -0.9 | -0.7 | 1.05 | 1.12 |
| *PSME3* | -0.8 | -0.55 | -0.81 | 1.27 | 0.89 |
| *CCT8* | -0.63 | -0.7 | -0.84 | 1.28 | 0.88 |
| *ZSCAN25* | -0.64 | -0.72 | -0.79 | 1.34 | 0.81 |
| *DEK* | -0.64 | -0.67 | -0.78 | 1.48 | 0.6 |
| *SLC39A8* | -0.72 | -0.83 | -0.55 | 1.41 | 0.69 |
| *ELP3* | -0.61 | -0.8 | -0.72 | 1.39 | 0.74 |
| *ZNF205* | -1.01 | -0.78 | -0.33 | 1.07 | 1.05 |
| *PSMA4* | -0.97 | -0.69 | -0.49 | 1.22 | 0.93 |
| *GIGYF2* | -1.03 | -0.66 | -0.43 | 1.27 | 0.84 |
| *EIF4B* | -0.79 | -0.76 | -0.64 | 1.17 | 1.02 |
| *RNF185* | -0.73 | -0.89 | -0.56 | 1.11 | 1.07 |
| *MEN1* | -0.82 | -0.84 | -0.51 | 1.06 | 1.11 |
| *SAP130* | -0.79 | -0.85 | -0.54 | 1.1 | 1.08 |
| *TYK2* | -0.7 | -0.93 | -0.51 | 1.28 | 0.87 |
| *RREB1* | -0.65 | -0.96 | -0.56 | 1.19 | 0.97 |
| *HMGA1* | -0.81 | -0.99 | -0.31 | 1.15 | 0.97 |
| *NRF1* | -0.82 | -0.97 | -0.31 | 1.26 | 0.84 |
| *ETV4* | -0.53 | -0.32 | -1.18 | 1.31 | 0.71 |
| *CDK14* | -0.66 | -0.23 | -1.15 | 0.77 | 1.27 |
| *CHGB* | -0.91 | 0.16 | -1.16 | 0.91 | 0.99 |
| *SF1* | -1.04 | -0.17 | -0.86 | 1.1 | 0.97 |
| *BAIAP2L2* | -1.04 | 0 | -0.96 | 1.03 | 0.97 |
| *BICDL1* | -0.74 | -0.33 | -1.05 | 1.07 | 1.05 |
| *CKAP2* | -0.69 | -0.28 | -1.12 | 1.01 | 1.09 |
| *TSN* | -0.73 | -0.26 | -1.09 | 1.19 | 0.89 |
| *SETD5* | -0.85 | -0.3 | -0.97 | 1.11 | 1.01 |
| *NR4A1* | -1 | -0.37 | -0.77 | 1.07 | 1.07 |
| *PDIA5* | -1.04 | -0.36 | -0.72 | 1.1 | 1.03 |
| *WRNIP1* | -1.08 | -0.34 | -0.7 | 1.06 | 1.06 |
| *RPS3* | -0.99 | -0.43 | -0.71 | 1.19 | 0.95 |
| *RNF123* | -0.9 | -0.44 | -0.8 | 1.21 | 0.94 |
| *AKR1B10* | -0.9 | -0.39 | -0.85 | 1.17 | 0.97 |
| *EIF1* | -1.08 | -0.4 | -0.55 | 0.62 | 1.41 |
| *CCT3* | -1.37 | -0.12 | -0.46 | 0.93 | 1.02 |
| *GRAMD1A* | -1.27 | -0.32 | -0.44 | 1.07 | 0.96 |
| *VIM* | 1.23 | 0.88 | -0.32 | -0.82 | -0.97 |
| *FN1* | 0.36 | 0.49 | 1.15 | -0.6 | -1.4 |
| *INO80* | 0.56 | 0.69 | 0.78 | -0.49 | -1.54 |
| *EMC1* | 0.72 | 0.66 | 0.76 | -0.77 | -1.37 |
| *IFI6* | 0.53 | 0.84 | 0.76 | -0.78 | -1.35 |
| *NDRG3* | 0.86 | 0.96 | 0.29 | -0.94 | -1.17 |
| *SCAMP1* | 0.52 | 1.08 | 0.51 | -0.89 | -1.22 |
| *ELOVL5* | 0.51 | 1.18 | 0.37 | -0.84 | -1.22 |
| *MAN2B1* | 0.93 | 0.62 | 0.46 | -0.51 | -1.51 |
| *EZH2* | 0.55 | 0.98 | 0.47 | -0.49 | -1.51 |
| *RASAL2* | 0.68 | 0.85 | 0.49 | -0.49 | -1.53 |
| *KDM1A* | 0.64 | 0.75 | 0.6 | -0.43 | -1.57 |
| *SLC29A4* | 0.6 | 0.83 | 0.58 | -0.47 | -1.55 |
| *PEX5* | 0.57 | 0.79 | 0.74 | -1.44 | -0.66 |
| *PRNP* | 0.47 | 0.77 | 0.88 | -1.36 | -0.75 |
| *MAP1LC3B* | 0.87 | 0.41 | 0.75 | -1.48 | -0.55 |
| *LSS* | 0.72 | 0.43 | 0.92 | -1.43 | -0.65 |
| *GNPAT* | 0.8 | 0.59 | 0.66 | -1.52 | -0.52 |
| *SEPT6* | 0.88 | 0.61 | 0.59 | -1.44 | -0.65 |
| *CSRP1* | 0.89 | 0.72 | 0.52 | -1.33 | -0.81 |
| *GTF3C3* | 0.8 | 0.64 | 0.71 | -1.31 | -0.85 |
| *ANKRD42* | 1.03 | 0.52 | 0.6 | -0.98 | -1.16 |
| *ZFP28* | 1.09 | 0.4 | 0.64 | -1.06 | -1.06 |
| *DNAJC10* | 1.08 | 0.68 | 0.33 | -1.21 | -0.88 |
| *ARHGEF3* | 1.07 | 0.79 | 0.23 | -1.03 | -1.06 |
| *UBE2O* | 0.64 | 0.54 | 0.97 | -1.13 | -1.03 |
| *RTN4RL2* | 0.58 | 0.64 | 0.93 | -0.91 | -1.25 |
| *DAPK1* | 0.62 | 0.76 | 0.81 | -1.02 | -1.16 |
| *TBC1D8* | 0.55 | 1.01 | 0.59 | -1.1 | -1.05 |
| *WDR41* | 0.5 | 0.9 | 0.76 | -1.16 | -1.01 |
| *POGZ* | 0.83 | 0.72 | 0.61 | -0.94 | -1.23 |
| *ADRM1* | 0.7 | 0.81 | 0.67 | -0.97 | -1.21 |
| *DVL3* | 0.75 | 0.73 | 0.71 | -1.02 | -1.17 |
| *PCNA* | 0.75 | 0.82 | 0.61 | -1.12 | -1.06 |
| *C16orf72* | 0.84 | 0.68 | 0.67 | -1.07 | -1.12 |
| *HUWE1* | 0.82 | 0.6 | 0.75 | -1.16 | -1.03 |
| *UCA1* | 1.49 | -0.04 | 0.37 | -0.89 | -0.92 |
| *LRRFIP2* | 1.18 | 0.26 | 0.6 | -1.22 | -0.83 |
| *ANKRD1* | 0.91 | 0.31 | 0.88 | -1.28 | -0.82 |
| *XAGE1A* | 0.95 | 0.27 | 0.9 | -1.07 | -1.04 |
| *DNAJC16* | 1.18 | 0.05 | 0.74 | -0.7 | -1.25 |
| *SLC2A3* | 0.78 | 0.19 | 1.08 | -0.83 | -1.22 |
| *CFTR* | 0.93 | 0.07 | 1.03 | -0.94 | -1.09 |
